# Mechanistic evaluation of ertugliflozin in patients with type 2 diabetes and heart failure

**DOI:** 10.14814/phy2.70275

**Published:** 2025-04-10

**Authors:** Yuliya Lytvyn, Rosalie A. Scholtes, Eva M. Boorsma, Vikas S. Sridhar, Luxcia Kugathasan, Hongyan Liu, Leif E. Lovblom, Louis Handoko, Charlotte M. Mosterd, John S. Floras, Kevin Burns, Tosin Osuntokun, Adriaan Voors, Daniel H. van Raalte, Hiddo J. L. Heerspink, David Z. I. Cherney

**Affiliations:** ^1^ Division of Nephrology, Department of Medicine University Health Network Toronto Ontario Canada; ^2^ Temerty Faculty of Medicine University of Toronto Toronto Ontario Canada; ^3^ Department of Internal Medicine, Diabetes Center Amsterdam UMC Amsterdam The Netherlands; ^4^ Department of Clinical Pharmacy and Pharmacology University of Groningen, University Medical Center Groningen Groningen The Netherlands; ^5^ Department of Cardiology, University Medical Center Groningen University of Groningen Groningen The Netherlands; ^6^ Biostatistics Department University Health Network Toronto Ontario Canada; ^7^ Institute of Health Policy, Management and Evaluation University of Toronto Toronto Ontario Canada; ^8^ Division of Cardiology University Health Network Toronto Ontario Canada; ^9^ Kidney Research Centre, The Ottawa Hospital University of Ottawa Ottawa Ontario Canada

**Keywords:** ertugliflozin, heart failure, SGLT2 inhibition, type 2 diabetes

## Abstract

The effect of sodium‐glucose cotransporter‐2 (SGLT2) inhibitor ertugliflozin on fluid volume and kidney function was assessed in patients with type 2 diabetes and heart failure. Thirty‐four participants were randomized in this double‐blind, placebo‐controlled, parallel‐group, multicenter study. Physiologic measurements were obtained under clamped euglycemia at baseline, 1 week, and 12 weeks of treatment. The primary outcome was the proximal tubular natriuretic effect of ertugliflozin versus placebo, measured by fractional excretion of lithium (FELi). Ertugliflozin did not increase FELi or total FENa at 1 week or 12 weeks. Ertugliflozin increased both mean 24‐h urinary sodium excretion (47.5 ± 22.1 mmol/day vs. placebo, *p* = 0.032) and urinary volume (*p* = 0.009) at 1 week, which was attenuated at Week 12. Reductions in extracellular fluid (−1.9 ± 0.8 L, *p* = 0.01), estimated plasma volume (−11.9 ± 13.9%, *p* = 0.02), and supine mean arterial pressure (−6.6 ± 2.7 mmHg, *p* = 0.02) were significant at Week 12. Compared to placebo, ertugliflozin acutely increased circulating angiotensinogen and angiotensin‐converting enzyme (ACE) levels, as well as urine adenosine and ACE2 activity (*p* < 0.05). Changes in other neurohormones, sympathetic activity, kidney, and systemic hemodynamics did not differ compared to placebo. Our findings suggest that SGLT2 inhibition shifts systemic volume toward a state of euvolemia, potentially lowering the risk of worsening heart failure.

## Graphical abstract

HF patients exhibit counterregulatory adaptations to tubular sodium handling. SGLT2 inhibition shifts systemic volume toward a state of euvolemia, potentially lowering the risk of worsening HF.

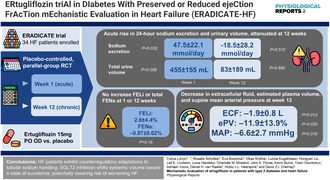

## 1 INTRODUCTION

Patients with type 2 diabetes (T2D) are at high risk of being hospitalized for heart failure (HF). Despite treatment with renin–angiotensin–aldosterone system (RAAS) inhibitors, mineralocorticoid receptor antagonists, and beta‐blockers, HF is associated with significant morbidity, with a mortality rate of 50% within 5 years of diagnosis (Nair, 2020). Importantly, clinical trials have demonstrated that sodium‐glucose co‐transporter 2 (SGLT2) inhibitors reduce mortality and HF risk, as well as decrease the risk of progressive diabetic kidney disease (DKD) (Cannon et al., 2020; Neal et al., 2017; Zinman et al., 2015). Subsequent trials, such as DAPA‐HF, EMPEROR‐reduced, EMPEROR‐preserved, and DELIVER, demonstrated that SGLT2 inhibition reduces the combined risk of worsening HF or cardiovascular death in patients with reduced or preserved ejection fraction, regardless of type 2 diabetes status (McMurray et al., 2019; Packer et al., 2020; Solomon et al., 2022). As a result, SGLT2 inhibitors are part of guideline‐directed medical therapy in HF patients. Kidney and cardiovascular benefits have been attributed to glycemic‐independent physiological effects, including natriuretic properties leading to intrarenal and systemic vascular hemodynamic benefits. Despite clinical benefits observed in these pivotal trials, the factors responsible for the beneficial effects of SGLT2 inhibitors in patients with T2D with respect to HF remain incompletely understood.

In the earlier hyperfiltration stage of DKD, SGLT2 inhibitors impact intrarenal tubuloglomerular feedback (TGF) mechanisms, leading to an acute “dip” in eGFR on the basis of reductions in intraglomerular hypertension (Cherney et al., 2014; Lytvyn et al., 2022). Enhanced distal sodium and chloride delivery, leading to increased TGF, may at least partially mediate the beneficial effects of SGLT2 inhibitors. Furthermore, intrarenal physiological effects resulting in acute GFR dipping are closely associated with reductions in albuminuria and attenuated CKD progression. Increased diuresis, leading to both reduced glomerular pressure and protection against volume overload, represents a possible common physiological pathway that augments kidney and cardiovascular protection in people with HF. However, few, if any, human studies have included integrative physiological investigations using kidney, cardiovascular, and neurohormonal measurements—an approach that is critical to better understand how SGLT2 inhibitors benefit the cardiorenal axis.

Accordingly, our aim was to elucidate the mechanisms whereby ertugliflozin impacts factors that regulate fluid volume and neurohormonal activation in patients with T2D and HF. The primary objective was to determine if SGLT2 inhibitors cause a proximal tubular natriuresis leading to a subsequent reduction in plasma volume and extracellular body water after 1 week and 12 weeks. Our secondary objective was to determine if volume contraction impacts natriuretic hormones that are activated in HF patients, such as B‐type natriuretic peptides (BNPs). Additionally, we studied the impact of ertugliflozin on cardiorenal hemodynamics, including echocardiographic measures, arterial stiffness, and systemic vascular resistance. We hypothesized that ertugliflozin increases natriuresis, thereby reducing plasma volume, without inducing renal vasoconstriction or activation of the sympathetic nervous system (SNS).

## 2 MATERIALS AND METHODS

### 2.1 Study participants

Thirty‐four participants with HF and T2D taking standard HF therapies successfully completed the study and had the primary natriuresis end point evaluated (Figure S1). Study participants were recruited from outpatient HF clinics at hospitals based in the Netherlands (Amsterdam University Medical Center, University Medical Center Groningen) and Canada (Toronto). Detailed study inclusion/exclusion criteria at screening are documented in Table S1. In brief, inclusion criteria were as follows: male or female patients ≥18 years diagnosed with HF with New York Heart Association (NYHA) Class 2–3 symptoms, ejection fraction ≥20%, and T2D diagnosed ≥2 months prior to informed consent; HbA1c of 6.5%–10.5%; blood pressure ≤160/110 and ≥90/60 mmHg at screening; on a stable dose of a maximally tolerated angiotensin‐converting enzyme (ACE) inhibitor, angiotensin receptor blocker (ARB), or renin inhibitor for at least 30 days prior to screening; on a stable diuretic dose for ≥30 days prior to the physiological assessment; and BNP levels ≥100 pg/mL (≥200 pg/mL if in atrial fibrillation). Main exclusion criteria were as follows: Type 1 diabetes, untreated leukocyte and/or nitrite positive urinalysis, severe hypoglycemia within 2 months prior to screening, unstable coronary artery disease with acute coronary syndrome, percutaneous intervention or bypass surgery within 3 months, or congestive HF secondary to infiltrative cardiomyopathy or pericardial constriction.

### 2.2 Experimental design

The study was registered at www.clinicaltrials.gov (NCT03416270). This was a multicenter prospective, double‐blind, placebo‐controlled parallel assignment study comprising a screening visit and a 4‐week run‐in phase for those not on stable angiotensin‐converting enzyme inhibitor (ACEi) or ARB and diuretic treatment, followed by a 12‐week double‐blind treatment period (Figure S2). Patients who met all inclusion/exclusion criteria and were on a stable maximum tolerated dose of ACEis or ARBs for at least 4 weeks proceeded directly to randomization, while patients who had their ACEi or ARB up‐titrated to a maximum tolerated dose during the preceding 4 weeks of the screening visit proceeded to a run‐in phase during which the type and dose of these drugs were stabilized. Participants were randomized to either 15 mg oral ertugliflozin (Steglatro™; Merck Canada Inc.; DIN: 02475529) or a matched placebo in a 1:1 ratio.

Participants underwent a full day of cardiorenal physiology assessments under controlled euglycemic clamp conditions (4–6 mmol/L) (Cherney et al., 2008) during the following visits: (Nair, 2020) baseline at Week 0 prior to randomization, (Zinman et al., 2015) acute treatment after 1 week of therapy, and (Neal et al., 2017) chronic treatment visit after 12 weeks of therapy. Clamped euglycemia was maintained to reduce the background glycemic variability and the impact of hyperglycemia on outcome measures, such as increased hyperfiltration (Cherney et al., 2008; Miller, 1999), raised blood pressure, and neurohormonal activation. Participants were supine throughout the physiologic assessments but were allowed to ambulate for voiding. Participants were counseled to maintain a stable habitual dietary salt intake to avoid volume contraction and RAAS activation from sodium depletion. Participants were also asked to avoid alcohol and tobacco for at least 48 h and fast for a minimum of 12 h before all physiologic assessment study visits. The use of nonsteroidal anti‐inflammatory drugs (NSAIDS), systemic corticosteroids, and immunosuppressants was discouraged for 4 weeks prior to physiologic measurements as these medications may interfere with the evaluation of safety, tolerability, and/or efficacy. The doses of diuretics and vitamin D analogues were to be stable at least 4 weeks prior to enrollment. Study medication was taken during the study visits before physiological measurements were obtained.

### 2.3 Assessment of renal sodium handling

Proximal tubular sodium handling was assessed using established sodium and lithium clearance techniques (Koomans et al., 1989; Montanari et al., 2012). To measure lithium clearance, participants were instructed to take a single 300 mg lithium carbonate capsule (PMS‐lithium carbonate; Pharmascience Inc.; DIN: 02216140) at 22:00 h the night before physiologic study visits. Blood and urine samples were collected after euglycemia was achieved during the physiologic study visits for sodium, lithium, and creatinine measurements. The following formulae were used for calculations of tubular sodium handling (Montanari et al., 1988):

$$\mathrm{Na}\;\text{Clearance}\;\left(C_{\mathrm{Na}}\right)=\frac{\left[\text{Urine}\;\mathrm{Na}\right]\times \text{Urine flow rate}}{\left[\text{Plasma}\;\mathrm{Na}\right]}$$

$$\mathrm{Li}\;\text{Clearance}\;\left(C_{\mathrm{Li}}\right)=\frac{\left[\text{Urine}\;\mathrm{Li}\right]\times \text{Urineflow rate}}{\left[\text{Plasma}\;\mathrm{Li}\right]}$$

$${\text{Absolute}}^{“}{\text{proximal}}^{”}\;\text{fluid}\;\text{reabsorption rate}\;\left(\mathrm{APR}\right)=\mathrm{GFR}-C_{\mathrm{Li}}$$

$${\text{Absolute}}^{“}{\text{proximal}}^{”}\;\mathrm{Na}\;\text{reabsorption rate}\;\left({\mathrm{APR}}_{\mathrm{Na}}\right)=\text{Plasma}\;\mathrm{Na}\;x\;\mathrm{APR}$$

$${\text{Fractional}}^{“}{\text{proximal}}^{”}\;\text{fluid reabsorption}\;\left(\mathrm{FPR}\right)=\frac{\mathrm{APR}}{\mathrm{GFR}}$$

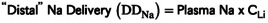

$$\begin{array}{c} {\text{Absolute}}^{“}{\text{Distal}}^{”}\;\mathrm{Na}\;\text{Reabsorption}\;\text{rate}\;\left({\mathrm{ADR}}_{\mathrm{Na}}\right)=\\ {\mathrm{DD}}_{\mathrm{Na}}-\left(\text{Urine}\;\mathrm{Na}\times \text{Urine flow rate}\right) \end{array}$$

$${\text{Fractional}}^{“}{\text{Distal}}^{”}\;\mathrm{Na}\;\text{Reabsorption}\;\left({\mathrm{FDR}}_{\mathrm{Na}}\right)=\frac{{\mathrm{ADR}}_{\mathrm{Na}}}{{\mathrm{DD}}_{\mathrm{Na}.}}$$

FeNa and FeLi calculations were conducted using the conventional formula as follows:

$${\mathrm{FE}}_{\left[\text{electrolyte}\right]}=\frac{\left[\text{urine electrolyte}\right]\times \left[\text{plasma creatinine}\right]}{\left[\text{plasma electrolyte}\right]\times \left[\text{urine creatinine}\right]}$$

### 2.4 Assessment of kidney hemodynamic function

After stabilization of the ambient euglycemic clamp, a third intravenous line was used to infuse iohexol (Omnipaque® 300; GE Healthcare Canada Inc.; DIN: 02172747) and para‐aminohippurate (PAH 20% solution; Bachem Distribution Services, GmbH, Weil am Rhein, Germany) (Zannad et al., 2022), as described previously (Cherney et al., 2014). Glomerular filtration rate (GFR) and effective renal plasma flow (ERPF) were estimated under steady‐state conditions of infusing iohexol and PAH, respectively (Cherney et al., 2008, 2014). Mean baseline GFR and ERPF values were calculated as a mean of two independent clearance periods on each of the physiology assessment visits. The following parameters were calculated:

$$\text{Filtration fraction}\;\left(\mathrm{FF}\right)=\frac{\mathrm{GFR}}{\text{ERPF}}$$

$$\text{Renal blood flow}\;\left(\mathrm{RBF}\right)=\frac{\text{ERPF}}{1-\text{Hematocrit}}$$

$$\text{Renal vascular resistance}\;\left(\mathrm{RVR}\right)=\frac{\mathrm{MAP}}{\mathrm{RBF}}$$

Mean arterial pressure (MAP, mmHg), ERPF (mL/s), GFR (mL/s), and total protein (g/dL) were used to indirectly estimate efferent (*R*_*E*_, dyne sec cm^−5^) and afferent arteriolar resistances (*R*_*A*_, dyne sec cm^−5^), and glomerular hydrostatic pressure (*P*_GLO_, mmHg) using Gomez's equations (Gomez, 1951). Assumptions imposed by Gomez's equations were the following: (i) intrarenal vascular resistances are divided into afferent, postglomerular, and efferent; (ii) hydrostatic pressures within the renal tubules, venules, Bowman's space, and interstitium (*P*_Bow_) are in equilibrium at 10 mmHg; (iii) glomerulus is in filtration disequilibrium; and (iv) the gross filtration coefficient (*K*_FG_) is 0.1012 mL/s per mmHg (*P*_GLO_ = 56.4 mmHg) for patients with T2D to reflect the different *P*_GLO_ values in diabetic and control conditions observed in previous micropuncture studies in Munich‐Wistar rats (Hostetter et al., 1981).

The filtration pressure across glomerular capillaries (Δ*P*_*F*_) is as follows:

$$\Delta P_{F}\;=\mathrm{GFR}/K_{\mathrm{FG}}$$

The *π*_*G*_ from the plasma protein mean concentration (*C*_*M*_) within the capillaries is as follows:

$$C_{M}=\mathrm{TP}/\mathrm{FF}\times \ln\left(1/1-\mathrm{FF}\right)$$

$$\pi_{G}=5\times \left(C_{M}-2\right)$$

Glomerular hydrostatic pressure (*P*_GLO_) is as follows:

$$P_{\mathrm{GLO}}=\Delta P_{F}+P_{\mathrm{Bow}}+\pi_{G}$$

*R*_*A*_ and *R*_*E*_ were estimated using principles of Ohm's law, where 1328 is the conversion factor to dyne sec cm^−5^ (Gomez, 1951):

$$R_{A}=\left[\left(\mathrm{MAP}-P_{\mathrm{GLO}}\right)/\mathrm{RBF}\right]\times 1328$$

$$R_{E}=\left[\mathrm{GFR}/K_{\mathrm{FG}}\times \left(\mathrm{RBF}-\mathrm{GFR}\right)\right]\times 1328$$

### 2.5 Assessment of cardiovascular hemodynamic function and metabolic parameters

MAP, blood pressure, and heart rate were measured by an automated sphygmomanometer over the right brachial artery (DINAMAP® sphygmomanometer, Critikon, Florida, USA) throughout the physiologic assessment study days. During each physiologic assessment study day, following the glucose clamp, right radial artery and carotid waveforms were recorded with a high‐fidelity micromanometer, and using the validated transfer function, corresponding central aortic pressure waveform data were generated (SPC‐301, Millar Instruments SphygmoCor, AtCor Medical Systems Inc., Sydney, Australia). Augmentation index, an estimate of systemic arterial stiffness, was calculated as the difference between the second systolic peak and inflection point, expressed as a percentage of the central pulse pressure corrected to an average heart rate of 75 beats per minute. The aortic pulse wave velocity (PWV) was measured using the same device by sequentially recording electrocardiogram‐gated right carotid and radial artery waveforms. The average of two vascular measurements was reported.

After completion of arterial stiffness testing, heart rate variability testing was performed using AtCor software (Atcor Medical Systems Inc., Sydney, Australia). The average of two 10‐minute segments was recorded. Vagal tone (root mean square successive difference [RMSSD]) and sympathetic measures (standard deviation of normal‐to‐normal interval [SDNN]) were obtained at each of the two periods, and the results were then averaged (Lytvyn et al., 2017, 2022).

Noninvasive cardiac output monitoring (NICOM, Cheetah Medical, Newton Center, MA, USA) was used to measure cardiac output, cardiac power output index, stroke volume, total peripheral resistance, total peripheral resistance index, and thoracic fluid content during each of the physiologic assessment study visits. The NICOM monitoring system is a form of bioelectrical impedance (“bioreatance”) to measure thoracic fluid content with electrocardiogram electrode stickers. Four sensor pads were applied above and below the heart on the chest. Each sensor pad contained an outer transmitting sensor and an inner receiving sensor. The NICOM monitor induced a 75‐KHz AC current to the thorax via the outer sensors and received the voltage via the inner sensors. The sensors can detect a time delay or phase shift between the induced current and the received voltage. Beat‐to‐beat changes in fluid content were used to derive cardiac output. Then, using measured MAP, systemic vascular resistance was calculated. Stroke volume was derived based on consecutive measurements of the phase shift. NICOM measurements were performed for 10 minutes, and in duplicate, the mean of the measurements was reported. Weight, waist circumference, fasting plasma glucose, and HbA1c were measured during each physiologic assessment study visit.

Transthoracic 2D echocardiography image acquisition was performed via a General Electrics Vivid 7 and E9 (GE, General Electric Corp, Wauwatosa, WI) ultrasound system. The transducer (2.5–5 MHz, Vivid7 pro ultrasound equipment, GE Health, Milwaukee, WI, USA) was used to allow adequate penetration for endocardial border definition. The same transducer was used for all visits for each given patient. Patients were examined at rest in the left lateral position during sinus rhythm. At least three cardiac cycles were recorded at each visit with the following views: parasternal long axis view, parasternal RV inflow view, apical four‐chamber view, apical five‐chamber view, and subcostal view. Left ventricular ejection fraction was measured using Simpson's biplane method. Plasma volume was measured using a nonradioactive technique (indocyanine green dilution) to assess changes in plasma volume in response to SGLT2 inhibition. Plasma volume was also estimated using the Strauss equation (Duarte et al., 2015; Strauss et al., 1951). Extracellular water was measured noninvasively using bioimpedance spectroscopy.

### 2.6 Sample collection and analytical methods for plasma and urine biochemistry responses

During the steady‐state euglycemic clamp conditions, iohexol and PAH blood samples were collected to assess renal clearance parameters according to standard methods (Cherney et al., 2014; Lytvyn et al., 2017). In addition, blood samples for angiotensin II, angiotensinogen, aldosterone, plasma renin concentration, ACE protein, ACE2 protein, plasma renin activity (PRA), BNP, NT‐proANP, norepinephrine, and epinephrine were also collected to determine the impact of treatment interventions on these parameters (Burns et al., 2017; Cherney et al., 2014; Lytvyn et al., 2017, 2019; Xiao & Burns, 2017). At corresponding time intervals, urine samples were collected to assess nitric oxide (NO), angiotensin II, angiotensinogen, adenosine, ACE protein, ACE activity, ACE2 protein, ACE2 activity, and NT‐proANP (Lytvyn et al., 2017). All urinary values were corrected for urinary creatinine at the time of collection and were expressed as a ratio relative to the amount of urinary creatinine excreted at the collection time points. In the 24 h leading up to physiological assessments, participants also completed a 24‐h timed urine collection, which was analyzed for volume, glucose excretion, urea, potassium, sodium, protein, albumin, 8‐isoprostance, 8‐OHdG, cGMP, nitrate, nitrite, and PGF2α.

Plasma NT‐proANP (Catalog No. MBS2513493), BNP (Catalog No. HCVD1MAG‐67 K), cGMP (Catalog No. MBS160025), NO (Catalog No. KGE001), PGF2α (Catalog No. MBS763933), 8‐hydroxydeoxyguanosine (Catalog No. ab201734), 8‐isoprostane (Catalog No. ab175819), angiotensin II (Catalog No. MBS703599), ACE2 substrate (Catalog No. 60757[AN]), and angiotensinogen (Catalog No. DY3156‐05) assessments were performed based on the ELISA kit instructions provided by the manufacturer (R&D Systems, Minneapolis, MN). Plasma renin concentrations were measured with a sandwich chemiluminescence immunoassay kit (Catalog No. M0870004217; LIAISON®, DiaSorin S.p.A., Italy) and enzymatic assays, including PRA (Catalog No. MBS495044), ACE (Catalog No. MBS9359476), and ACE2 activity (Catalog No. DY933‐05), were measured using ELISA kits. Aldosterone, catecholamines, and urine adenosine were measured using chromatography and mass spectrometry, while ELISA was used to measure urine cGMP (Catalog No. MBS160025), 8‐isoprostane (Catalog No. ab175819), NO (Catalog No. KGE001), angiotensin II (Catalog No. MBS703599), and angiotensinogen (Catalog No. DY3156‐05) levels (R&D Systems, Minneapolis, MN). Urine lithium was measured by atomic absorption, and 24‐h urine glucose was assessed by standard laboratory methods. Urine and plasma sodium and creatinine were measured using the Abbott Alinity I Immunology Analyzer instrument. HbA_1c_ was measured by high‐performance liquid chromatography.

### 2.7 Statistical analyses

The primary end point of this study was the difference in proximal fractional sodium excretion, measured using fractional excretion of lithium (FE_Li_) as a surrogate after a 12‐week treatment with ertugliflozin compared to placebo. Based on preclinical studies and assuming a baseline FE_Li_ of 22 ± 7, to be able to detect a 40% increase in FE_Li_ with 80% power and a two‐tailed alpha of 0.05, 18 patients per group were required, for a total sample size of 36 patients. Due to the restrictions and delays with the COVID‐19 pandemic, a total of 34 patients were randomized in the study.

To assess the primary outcome, linear mixed‐effects models were used. Covariates included visit, treatment group, and the visit‐by‐treatment group interaction; the vector of outcomes included the baseline value, and the interaction regression coefficient was used to determine the effect of ertugliflozin. Within‐group changes were also determined. The intention‐to‐treat population was used. To account for repeated measures within study participants, a participant‐level random intercept was defined and a compound symmetry covariance structure was assumed. Restricted maximum likelihood was used for estimation, the missing‐at‐random framework was assumed, and plots of marginal and conditional residuals were used for model diagnostics and assessment. To determine acute effects, this analysis was repeated using the baseline and 1‐week values only. Significant tests were based on a two‐sided α value of 0.05. Within‐group changes were also assessed, and all secondary outcomes were analyzed in the same manner. For the secondary outcomes, nominal p‐values were reported.

## 3 RESULTS

### 3.1 Baseline clinical characteristics

Thirty‐four participants with T2D and HF were enrolled and randomized to ertugliflozin (*n* = 17) or placebo (*n* = 17). Baseline characteristics were similar between both ertugliflozin and placebo groups (Table 1). In all, 42% of participants were male with a mean estimated GFR (eGFR) of 64.9 ± 22.6 mL/min/1.73 m^2^ and a urine albumin to creatinine ratio (UACR) of 22.1 ± 32.6 mg/mmol. All but one participant were concomitantly treated with diuretic therapy during the study. Diuretic dosages remained stable throughout the treatment period with a mean equipotent oral furosemide dose of 53.1 ± 45.3 mg in the ertugliflozin group and 90.7 ± 74.4 mg in the placebo group.

**TABLE 1:** Baseline demographic and clinical characteristics at randomization in participants with type 2 diabetes and heart failure.

	Ertugliflozin (*n* = 17)	Placebo (*n* = 17)	*p*‐Value
Male	15 (88)	13 (76)	0.66
Age, years	69.8 ± 8.7	69.4 ± 7.5	0.60
Ethnicity			1.00
Caucasian	13 (76)	12 (71)	
Black	1 (6)	0 (0)	
Asian	1 (6)	2 (12)	
Other	2 (12)	3 (18)	
Weight, kg	91.8 ± 19.2	96.9 ± 16.3	0.38
BMI, kg/m^2^	30.5 ± 6.5	32.4 ± 4.4	0.15
Waist circumference, cm	109.4 ± 16.9	117.3 ± 11.1	0.11
HbA1c, %	8.2 ± 1.1	7.6 ± 1.1	0.097
Hemoglobin, g/L	126.2 ± 16.4	131.1 ± 21.3	0.40
Glucose, mmol/L	8.6 ± 3.1	7.3 ± 1.8	0.33
SBP, mmHg	134.3 ± 22.5	129.9 ± 20.6	0.54
DBP, mmHg	77.9 ± 8.8	76.1 ± 12.4	0.40
HR, bpm	72.2 ± 11.7	74.2 ± 15.4	0.58
Ejection fraction, %	39.2 ± 15.5	40.4 ± 15.4	0.44
UACR, mg/mmol	16.3 ± 29.0	27.8 ± 35.8	0.067
eGFR, mL/min/1.73 m^2^	63.5 ± 23.3	66.4 ± 22.4	0.84
Concomitant medication			
ACEi/ARB	13 (76)	15 (88)	0.66
Diuretics	16 (94)	17 (100)	1.00
Loop diuretics	13 (76)	15 (88)	
Dose, mg^a^	53.1 ± 45.3	90.7 ± 74.4	
Thiazide diuretics	2 (11)	1 (6)	
Beta‐blockers	14 (82)	16 (94)	0.60
MRA	10 (59)	11 (65)	1.00

*Note*: Values are *n* (%) or mean ± SD. Waist circumference was measured at screening. Estimated glomerular filtration rate (eGFR) was estimated with the Chronic Kidney Disease Epidemiology Collaboration formula.

Abbreviations: ACEi, angiotensin‐converting enzyme inhibitor; ARB, angiotensin receptor blocker; BMI, body mass index; bpm, beats per minute; DBP, diastolic blood pressure; eGFR, estimated glomerular filtration rate; HR, heart rate; MRA, mineralocorticoid receptor antagonist; SBP, systolic blood pressure; UACR, urine albumin to creatinine ratio.

^a^ Furosemide oral dose equivalent.

### 3.2 Effects of ertugliflozin on renal tubular sodium handling

**FIGURE 1:**
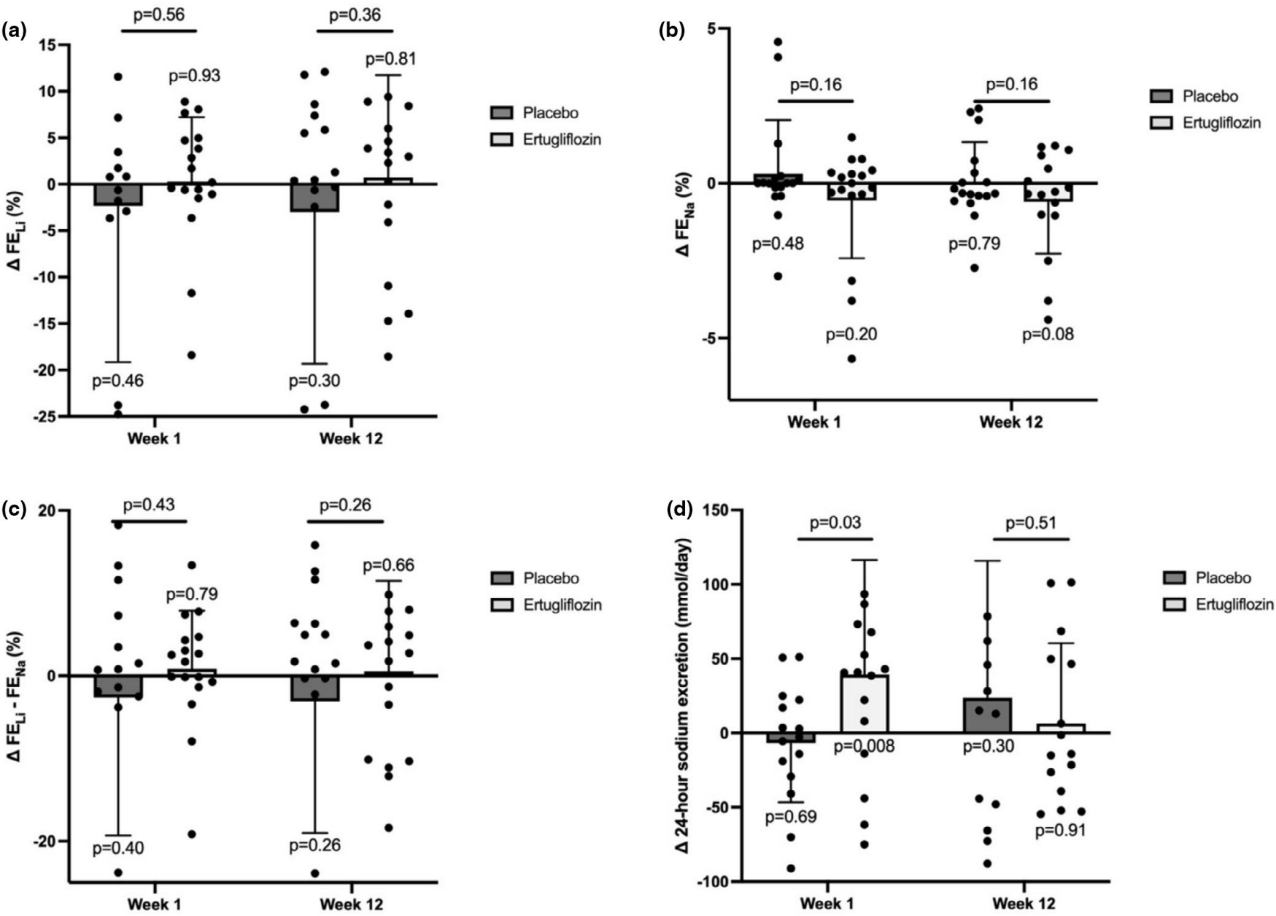
Acute and chronic changes in FE_Li_, FE_Na_, and absolute fractional distal sodium reabsorption in response to ertugliflozin compared with placebo in participants with type 2 diabetes and heart failure. Changes in (a) FE_Li_, (b) FE_Na_, (c) FE_Li_ − FE_Na_, and (d) 24‐h sodium excretion are calculated as differences in group means at Week 12 minus baseline (Week 0) and Week 1 minus baseline.

Ertugliflozin did not induce significant changes in proximal sodium excretion as determined by change in exogenous lithium clearance compared to placebo from baseline to 1 week (placebo‐adjusted mean ± standard error change FE_Li_ 2.6 ± 4.4%, *p* = 0.56) and 12 weeks (placebo‐adjusted mean ± standard error change FE_Li_ 4.5 ± 4.9%, *p* = 0.36) (Figure 1a; refer to Table S2 for the individual group means at each time point). There was also no effect of ertugliflozin compared to placebo on FE_Na_ from baseline to 1 week (placebo‐adjusted FE_Na_ *−* 0.87 ± 0.62%, *p* = 0.16) or 12 weeks (placebo‐adjusted FE_Na_ *−* 0.72 ± 0.51%, *p* = 0.16) with ertugliflozin. Absolute fractional distal sodium reabsorption (FE_Li_ − FE_Na_) was also not altered with ertugliflozin vs. placebo at 1 week and 12 weeks. Moreover, the relative fractional distal sodium reabsorption ([FE_Li_−FE_Na_]/FE_Li_) did not change from baseline to 1 week (*p* = 0.11) or 12 weeks (*p* = 0.53) with ertugliflozin compared to placebo. The total 24‐h urine volume increased at 1 week with ertugliflozin compared with placebo (*p* = 0.009), but was no longer statistically significant at 12 weeks (*p* = 0.66). This was accompanied by a placebo‐adjusted increase in 24‐h sodium excretion by 47.5 ± 22.1 mmol/day (*p* = 0.032) at 1 week, without significant placebo‐adjusted changes after 12 weeks of ertugliflozin (Table 7). Overall, ertugliflozin did not significantly impact proximal or distal sodium reabsorption; however, it increased 24‐h urine volume and sodium excretion at 1 week, suggesting an initial acute diuretic effect that diminishes over time.

### 3.3 Effects of ertugliflozin on kidney hemodynamic function

**FIGURE 2:**
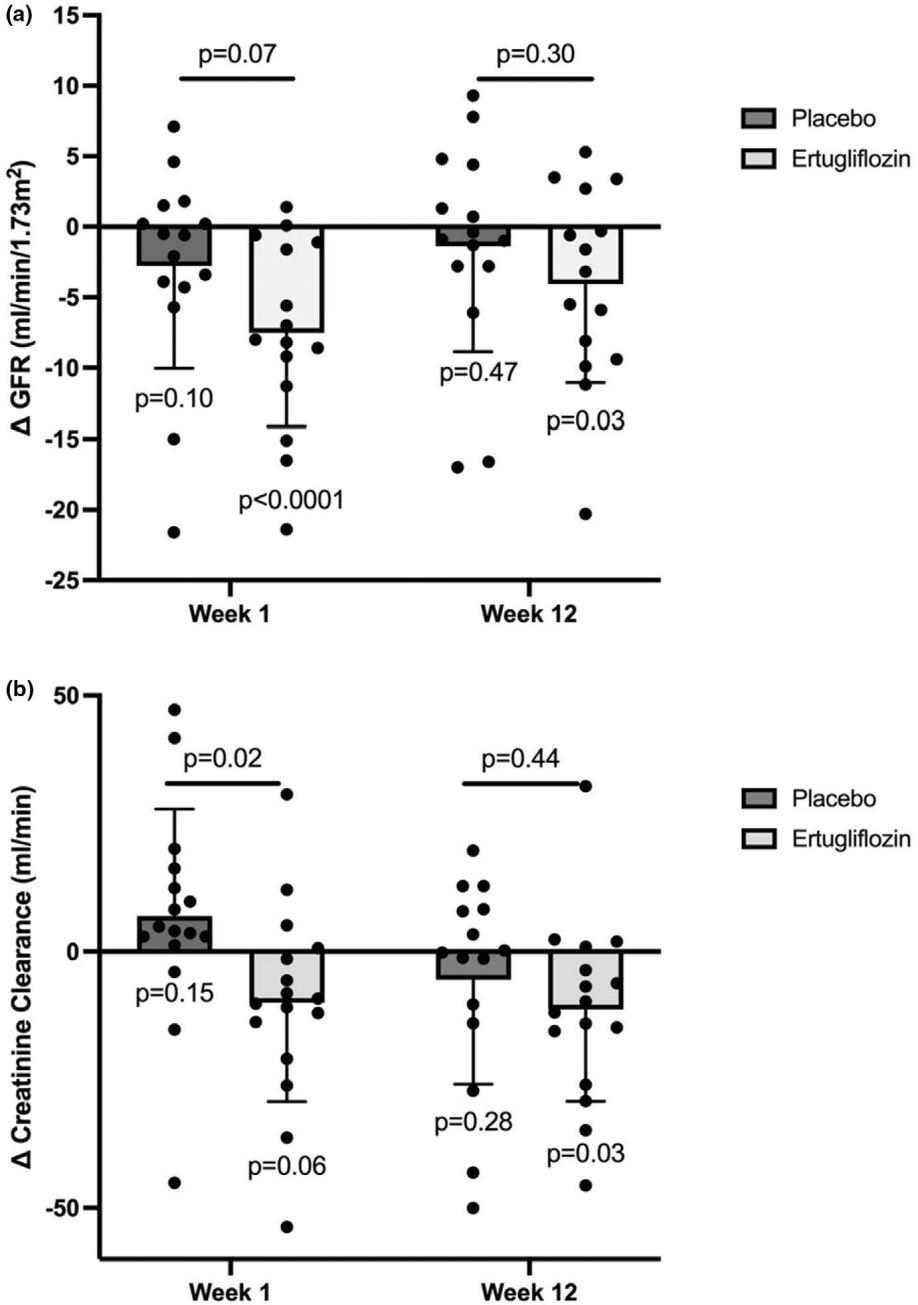
Changes in kidney hemodynamic measures with 1 week and 12 weeks of treatment in participants with type 2 diabetes and heart failure. Changes in (a) glomerular filtration rate (GFR) and (b) creatinine clearance during Week 1 and Week 12 of ertugliflozin treatment or placebo subtracted from baseline values.

Ertugliflozin significantly decreased iohexol‐measured GFR by 7.5 mL/min/1.73 m^2^ from baseline to 1 week (within‐group: 52.4 ± 4.1 to 44.9 ± 4.1 mL/min/1.73 m^2^, p_within group_ <0.0001). This was not statistically different when compared to placebo (*p* = 0.072) (Table 2). There was also an acute dip in 24‐h urine creatinine clearance at 1 week compared to placebo (placebo‐adjusted change of −16.7 ± 7.1 mL/min/1.73 m^2^, *p* = 0.02). Within‐group iohexol‐measured GFR changes were still significant but attenuated after 12 weeks of ertugliflozin treatment by 4.0 mL/min/1.73 m^2^ (within‐group: 52.6 ± 4.0 to 48.6 ± 4.0 mL/min/1.73 m^2^, *p*_within group_ = 0.03), though this change was not different versus placebo (*p* = 0.30) (Figure 2a). There were no significant within‐group or placebo‐adjusted changes in other kidney hemodynamic measures at 1 week or 12 weeks. In summary, ertugliflozin significantly reduced iohexol‐measured GFR and 24‐h urine creatinine clearance at 1 week, potentially indicating an early impact on renal function.

**TABLE 2:** Kidney hemodynamic responses to ertugliflozin compared with placebo in participants with type 2 diabetes and heart failure.

	Ertugliflozin (*n* = 17), mean ± SE	Placebo (*n* = 17), mean ± SE	*p*‐Value placebo‐adjusted change from baseline
*Absolute measured values*			
mGFR, mL/min/1.73 m^2^			
Baseline	52.4 ± 4.1	51.4 ± 4.2	Ref
Week 1	44.9 ± 4.1	48.5 ± 4.2	0.072
Week 12	48.6 ± 4.0	50.1 ± 4.2	0.303
ERPF^a^, mL/min/1.73 m^2^			
Baseline	341.0 ± 32.5	325.3 ± 30.5	Ref
Week 1	305.4 ± 31.8	352.5 ± 32.0	0.096
Week 12	399.1 ± 46.8	335.6 ± 41.5	0.438
*Gomez‐derived values* ^a^			
RBF, mL/min/1.73 m^2^			
Baseline	553.3 ± 59.6	546.6 ± 56.2	Ref
Week 1	494.7 ± 58.4	590.3 ± 59.9	0.107
Week 12	677.0 ± 81.1	551.9 ± 74.6	0.284
RVR, mL/min/1.73 m^2^			
Baseline	0.194 ± 0.018	0.175 ± 0.017	Ref
Week 1	0.189 ± 0.018	0.169 ± 0.018	0.962
Week 12	0.162 ± 0.018	0.162 ± 0.017	0.423
Filtration fraction			
Baseline	0.156 ± 0.014	0.167 ± 0.013	Ref
Week 1	0.156 ± 0.014	0.139 ± 0.014	0.117
Week 12	0.137 ± 0.014	0.154 ± 0.013	0.722
*R*_*A*_, dyn s^−1^ cm^−5^			
Baseline	7740.0 ± 820.8	6484.6 ± 776.6	Ref
Week 1	6970.6 ± 808.3	6245.5 ± 815.6	0.478
Week 12	6351.6 ± 929.7	5460.5 ± 887.1	0.784
*R*_*E*_, dyn s^−1^ cm^−5^			
Baseline	1419.9 ± 137.9	1462.6 ± 128.5	Ref
Week 1	1414.6 ± 133.8	1254.5 ± 141.3	0.283
Week 12	1144.0 ± 133.5	1363.6 ± 122.2	0.371
P_GLO_, mmHg			
Baseline	47.5 ± 1.1	47.2 ± 1.1	Ref
Week 1	47.1 ± 1.1	46.5 ± 1.1	0.726
Week 12	46.0 ± 1.3	47.4 ± 1.2	0.174
*Creatinine‐derived values*			
24‐h creatinine, mol/day			
Baseline	8.60 ± 1.11	6.83 ± 1.10	Ref
Week 1	8.24 ± 1.10	8.72 ± 1.11	0.025
Week 12	7.53 ± 0.95	6.63 ± 0.98	0.352
Creatinine clearance, mL/min			
Baseline	55.3 ± 6.9	46.7 ± 6.8	Ref
Week 1	45.9 ± 6.8	54.0 ± 6.9	0.019
Week 12	45.0 ± 6.2	41.4 ± 6.4	0.445

*Note*: Values are in estimated means ± standard error of the mean. Statistical comparisons between placebo and ertugliflozin groups were performed using the Wilcoxon Rank Sum test.

Abbreviations: GFR, glomerular filtration rate; ERPF, effective renal plasma flow; *P*_GLO_, glomerular hydrostatic pressure; *R*_*A*_, afferent arteriolar resistance; RBF, renal blood flow; *R*_*E*_, efferent arteriolar resistance; RVR, renal vascular resistance.

^a^ Renal plasma flow was only captured in a subset of the cohort (*n* = 28). Analysis of ERPF and Gomez‐derived values was therefore limited to this subset of participants.

### 3.4 Effect of ertugliflozin on cardiovascular hemodynamic measures

**FIGURE 3:**
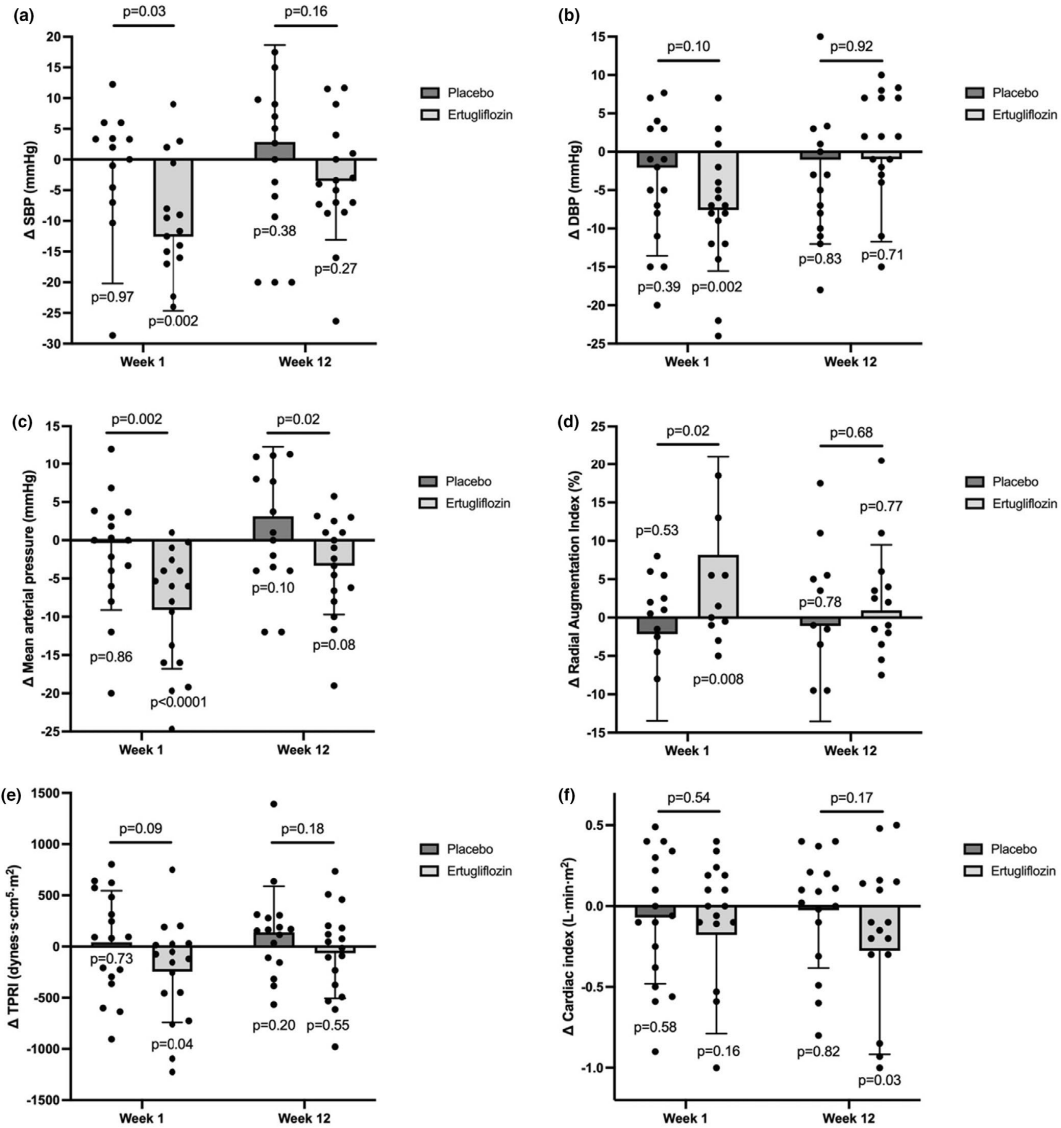
Changes in blood pressure, NICOM measures, and arterial stiffness after 1 week and 12 weeks of treatment. Changes in (a) systolic blood pressure (SBP) by NICOM, (b) clinical measure of diastolic blood pressure (DBP), (c) mean arterial pressure by NICOM, (d) radial augmentation index, (e) total peripheral resistance, and (f) cardiac index during Week 1 and Week 12 of ertugliflozin treatment or placebo subtracted from baseline values.

Acute treatment with ertugliflozin significantly reduced sitting diastolic blood pressure (DBP) by 7.6 mmHg (within‐group: 77.9 ± 2.4 to 70.3 ± 2.4 mmHg, *p*_within group_ = 0.002) at 1 week, but changes were not statistically different versus placebo at 1 week (*p* = 0.10) or 12 weeks (*p* = 0.92) (Figure 3). Supine measures of MAP measured during NICOM decreased by 8.8 ± 2.8 mmHg with ertugliflozin compared to placebo (*p* = 0.002) at 1 week. This was also accompanied by a placebo‐adjusted reduction in supine systolic blood pressure (SBP) of 12.5 ± 5.7 mmHg (*p* = 0.03). There were also within‐group reductions in total peripheral resistance index with ertugliflozin by 245 dynes·s·cm^5^·m^2^ (*p*_within group_ = 0.04) at 1 week, but this change was not statistically different compared with placebo at 1 week (*p* = 0.09) or 12 weeks (*p* = 0.18) (Table 3). Placebo‐adjusted reductions in supine MAP with ertugliflozin were still persistent at 12 weeks (*p* = 0.02), whereas no such effect was observed with supine SBP (*p* = 0.16). There were no significant within‐group or placebo‐adjusted changes in other seated or NICOM blood pressure measures at 1 week or 12 weeks (Table 3). Overall, while ertugliflozin had short‐term effects on blood pressure and vascular resistance, these changes were not largely sustained, indicating a transient response to treatment.

**TABLE 3:** Cardiovascular hemodynamic responses to ertugliflozin compared with placebo in participants with type 2 diabetes and heart failure.

	Ertugliflozin (*n* = 17), mean ± SE	Placebo (*n* = 17), mean ± SE	*p*‐Value placebo‐adjusted change from baseline
*Blood pressure*			
HR, bpm			
Baseline	72 ± 3	74 ± 3	Ref
Week 1	71 ± 3	71 ± 3	0.630
Week 12	74 ± 3	72 ± 3	0.405
SBP, mmHg			
Baseline	128 ± 4	127 ± 4	Ref
Week 1	127 ± 5	121 ± 5	0.294
Week 12	132 ± 5	128 ± 5	0.599
DBP, mmHg			
Baseline	78 ± 2	76 ± 2	Ref
Week 1	70 ± 2	74 ± 2	0.10
Week 12	77 ± 3	76 ± 3	0.92
MAP, mmHg			
Baseline	94 ± 3	92 ± 3	Ref
Week 1	88 ± 3	85 ± 3	0.978
Week 12	94 ± 4	89 ± 4	0.634
*Vascular measures of arterial stiffness*			
Radial augmentation index, %			
Baseline	*−*15.7 ± 3.9	*−*5.1 ± 4.5	Ref
Week 1	*−*6.7 ± 4.2	*−*7.3 ± 4.5	0.022
Week 12	*−*14.9 ± 3.5	*−*6.0 ± 4.0	0.684
Aortic augmentation index, %			
Baseline	23.2 ± 2.2	23.8 ± 2.3	Ref
Week 1	22.0 ± 2.2	24.1 ± 2.4	0.616
Week 12	22.2 ± 2.0	24.2 ± 2.2	0.580
Carotid augmentation index, %			
Baseline	22.3 ± 3.1	31.5 ± 2.8	Ref
Week 1	22.0 ± 3.1	30.7 ± 2.9	0.830
Week 12	24.7 ± 3.0	33.0 ± 2.9	0.829
Carotid radial pulse wave velocity, m/s			
Baseline	9.10 ± 0.57	8.85 ± 0.53	Ref
Week 1	8.93 ± 0.57	7.86 ± 0.57	0.369
Week 12	11.50 ± 1.43	8.70 ± 1.43	0.375
Carotid femoral pulse wave velocity, m/s			
Baseline	11.44 ± 1.10	10.52 ± 0.99	Ref
Week 1	10.89 ± 1.10	11.65 ± 1.04	0.258
Week 12	11.50 ± 1.50	12.14 ± 1.42	0.443
*Heart rate variability*			
RMSSD, ms			
Baseline	49.7 ± 15.4	41.0 ± 14.6	Ref
Week 1	60.5 ± 13.9	31.1 ± 14.6	0.422
Week 12	54.3 ± 13.5	38.9 ± 15.0	0.749
SDNN, ms			
Baseline	48.8 ± 10.9	37.7 ± 10.4	Ref
Week 1	44.8 ± 9.8	29.9 ± 10.3	0.844
Week 12	49.0 ± 10.8	36.2 ± 12.1	0.926
*NICOM*			
SBP, mmHg			
Baseline	137 ± 5	136 ± 5	Ref
Week 1	124 ± 5	136 ± 5	0.028
Week 12	133 ± 5	139 ± 5	0.160
DBP, mmHg			
Baseline	78 ± 3	76 ± 3	Ref
Week 1	71 ± 3	71 ± 3	0.745
Week 12	75 ± 3	79 ± 3	0.137
MAP, mmHg			
Baseline	98 ± 3	96 ± 3	Ref
Week 1	89 ± 3	96 ± 3	0.002
Week 12	94 ± 3	99 ± 3	0.016
Cardiac output, L/min			
Baseline	5.9 ± 0.3	6.0 ± 0.3	Ref
Week 1	6.0 ± 0.3	5.8 ± 0.3	0.534
Week 12	5.8 ± 0.3	5.8 ± 0.3	0.844
Cardiac index, L min m^2^			
Baseline	3.1 ± 0.1	2.8 ± 0.1	Ref
Week 1	2.9 ± 0.1	2.8 ± 0.1	0.545
Week 12	2.8 ± 0.1	2.8 ± 0.1	0.174
SV, mL/beat			
Baseline	90.1 ± 5.8	87.4 ± 5.8	Ref
Week 1	90.8 ± 5.8	87.5 ± 5.8	0.901
Week 12	89.3 ± 6.0	85.6 ± 6.1	0.836
SVV, %			
Baseline	15.8 ± 0.9	16.4 ± 0.9	Ref
Week 1	15.1 ± 0.9	16.0 ± 0.9	0.764
Week 12	17.2 ± 1.2	15.7 ± 1.2	0.316
SVI, mL m^2^ beat			
Baseline	43.8 ± 2.7	41.3 ± 2.7	Ref
Week 1	44.7 ± 2.7	41.9 ± 2.7	0.904
Week 12	43.2 ± 2.9	41.1 ± 2.9	0.880
TPR, dynes s cm^5^			
Baseline	1370.4 ± 95.6	1376.1 ± 95.6	Ref
Week 1	1261.0 ± 95.6	1404.6 ± 95.6	0.092
Week 12	1346.7 ± 99.1	1437.1 ± 99.9	0.262
TPRI, dynes s cm^5^ m^2^			
Baseline	2775.2 ± 192.6	2879.9 ± 192.6	Ref
Week 1	2530.5 ± 192.6	2921.7 ± 192.6	0.095
Week 12	2710.0 ± 203.9	3022.4 ± 205.5	0.180
CPO, mmHg L min			
Baseline	1.3 ± 0.1	1.2 ± 0.1	Ref
Week 1	1.2 ± 0.1	1.5 ± 0.1	0.133
Week 12	1.2 ± 0.1	1.3 ± 0.1	0.397
CPOI			
Baseline	0.63 ± 0.06	0.60 ± 0.06	Ref
Week 1	0.58 ± 0.06	0.69 ± 0.06	0.184
Week 12	0.61 ± 0.04	0.60 ± 0.04	0.576
TFC, 1/kΩ			
Baseline	39.2 ± 2.1	33.6 ± 2.1	Ref
Week 1	28.8 ± 2.1	33.5 ± 2.1	0.801
Week 12	39.7 ± 2.1	34.8 ± 2.1	0.708

*Note*: Values are in estimated means ± standard error of the mean. Statistical comparisons between placebo and ertugliflozin groups were performed using the Wilcoxon Rank Sum test.

Abbreviations: bpm, beats per minute; CPO, cardiac power output; CPOI, cardiac power output index; DBP, diastolic blood pressure; HR, heart rate; MAP, mean arterial pressure; NICOM, noninvasive cardiac output monitoring; RMSSD, root mean square successive difference; SBP, systolic blood pressure; SDNN, standard deviation of normal‐to‐normal interval; SV, stroke volume; SVI, stroke volume index; SVV, stroke volume variation; TFC, thoracic fluid content; TPR, total peripheral resistance; TPRI, total peripheral resistance index.

For echocardiographic parameters, compared to placebo at 1 week, ertugliflozin was associated with a decrease in indexed left ventricular stroke volume (placebo‐adjusted decrease of 4.9 ± 1.7 mL/m^2^, *p* = 0.005), but this did not sustain after 12 weeks of treatment (Table 4 and Figure S3). Despite no acute effects, treatment with ertugliflozin for 12 weeks compared to placebo significantly reduced the indexed left ventricular end‐diastolic volume by 12.1 ± 4.4 mL/m^2^ (*p* = 0.006). Cardiac index was also significantly decreased within the ertugliflozin group by 0.3 L·min·m^2^ (*p*_within group_ = 0.03), but no between‐group differences were seen compared with placebo at 1 week (p=0.54) or at 12 weeks (*p* = 0.17) (Figure 3 and Table 3). Likewise, the E/e′ ratio (*−*1.93 ± 0.98, *p*_within group_ = 0.049) was significantly changed with 12 weeks of ertugliflozin, but these changes were not statistically different compared to placebo (Table 4). Despite an acute, placebo‐adjusted increase in radial augmentation index that did not persist, no within‐group or placebo‐adjusted changes in echocardiographic or arterial stiffness measures were observed with ertugliflozin after 1 week or 12 weeks (Tables 3 and 4). In conclusion, these findings suggest that while ertugliflozin may have some short‐term effects on cardiac function and vascular stiffness, these effects are not sustained over the course of treatment.

**TABLE 4:** Cardiac remodeling in response to ertugliflozin compared with placebo in participants with type 2 diabetes and heart failure.

	Ertugliflozin (*n* = 17), mean ± SE	Placebo (*n* = 17), mean ± SE	*p*‐Value placebo‐adjusted change from baseline
LV EF, %			
Baseline	49.7 ± 4.6	41.9 ± 5.5	Ref
Week 1	50.8 ± 4.6	42.1 ± 5.5	0.608
Week 12	49.2 ± 4.4	43.6 ± 5.4	0.536
LV SV index, mL/m^2^			
Baseline	31.9 ± 1.9	25.3 ± 2.2	Ref
Week 1	30.6 ± 2.0	28.9 ± 2.2	0.005
Week 12	28.7 ± 2.6	29.3 ± 2.3	0.137
LAV index, mL/m^2^			
Baseline	41.6 ± 2.9	40.0 ± 3.1	Ref
Week 1	42.5 ± 3.0	40.8 ± 3.2	0.972
Week 12	37.5 ± 2.8	39.8 ± 2.9	0.133
LV ESV index, mL/m^2^			
Baseline	28.7 ± 5.3	41.3 ± 6.4	Ref
Week 1	26.0 ± 5.5	40.4 ± 6.3	0.565
Week 12	31.9 ± 5.4	43.1 ± 6.9	0.137
LV EDV index, mL/m^2^			
Baseline	63.0 ± 5.7	69.6 ± 7.4	Ref
Week 1	55.1 ± 6.9	69.0 ± 7.7	0.678
Week 12	57.4 ± 5.8	76.1 ± 7.4	0.006
E/e′			
Baseline	13.3 ± 1.1	12.2 ± 1.2	Ref
Week 1	12.4 ± 1.1	10.9 ± 1.2	0.799
Week 12	11.3 ± 1.2	13.6 ± 1.2	0.061

*Note*: Values are in estimated means ± standard error of the mean. Statistical comparisons between placebo and ertugliflozin groups were performed using the Wilcoxon Rank Sum test.

Abbreviations: E/e′, ratio of early diastolic velocity of mitral inflow to the early diastolic velocity of mitral annular motion; EDV, end diastolic volume; EF, ejection fraction; ESV, end systolic volume; LAV, left atrial volume; LV, left ventricle; LVOT, left ventricular outflow tract; SV, stroke volume.

### 3.5 Effects of ertugliflozin on plasma volume and body composition

**FIGURE 4:**
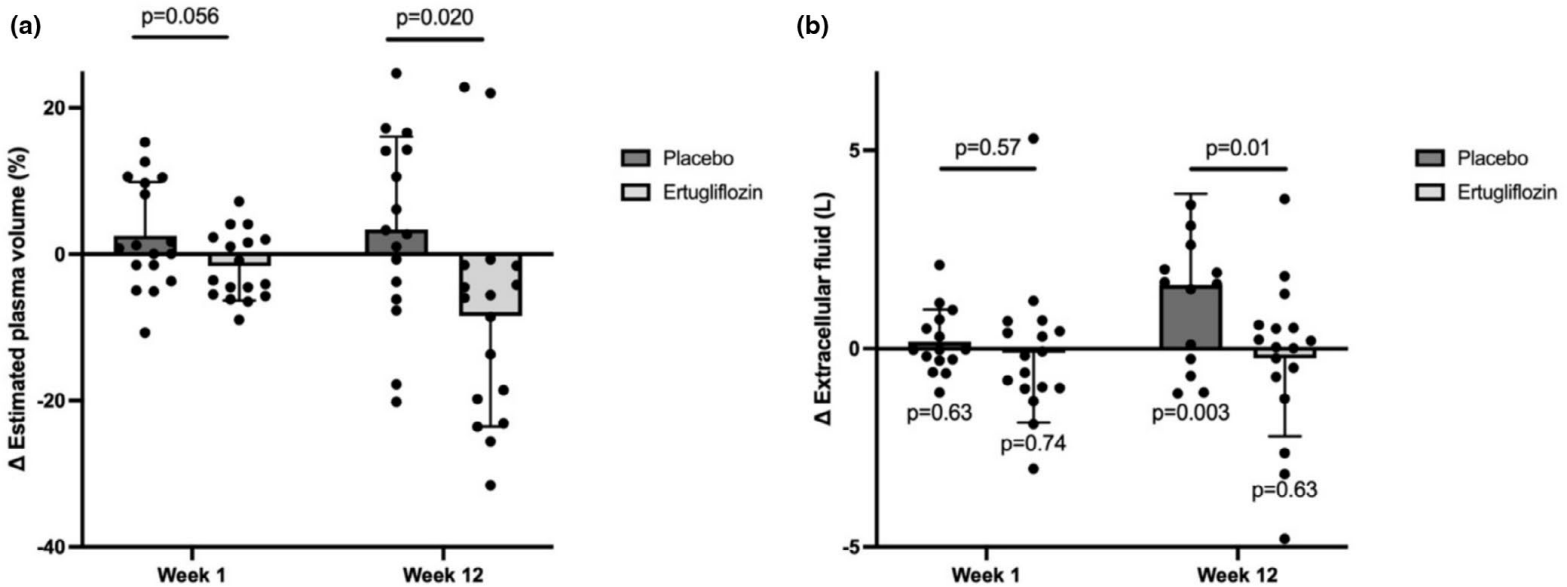
Changes in estimated plasma volume and extracellular fluid volume after 1 week and 12 weeks. Changes in (a) estimated plasma volume calculated by Strauss estimation and (b) extracellular fluid volume during Week 1 and Week 12 of ertugliflozin treatment or placebo subtracted from baseline values.

Compared to placebo, ertugliflozin treatment for 12 weeks lowered extracellular fluid (placebo‐adjusted decrease of 1.9 ± 0.8 L, *p* = 0.013), at least in part due to a rise in extracellular fluid in placebo‐treated participants (1.66 ± 0.57 L, *p*_within group_ = 0.003) (Table 5). Compared to placebo, ertugliflozin treatment for 12 weeks also decreased estimated plasma volume by 11.9 ± 13.9% (*p* = 0.02) (Figure 4). There were no other significant, placebo‐adjusted differences observed in the body composition analysis, including fat mass, fat‐free mass, or total body water outcomes. These results suggest that longer treatment with ertugliflozin led to volume contraction, particularly in extracellular and plasma compartments, without impacting broader body composition.

**TABLE 5:** Body composition and fluid levels in response to ertugliflozin compared with placebo in participants with type 2 diabetes and heart failure.

	Ertugliflozin (*n* = 17), mean ± SE	Placebo (*n* = 17), mean ± SE	*p*‐Value placebo‐adjusted change from baseline
Estimated plasma volume, %			
Week 1—baseline	*−*1.65 ± 4.66	2.54 ± 7.31	0.056
Week 12—baseline	*−*8.46 ± 15.08	3.39 ± 19.63	0.020
Extracellular fluid, L			
Baseline	20.8 ± 0.9	20.1 ± 1.0	Ref
Week 1	20.3 ± 0.9	20.3 ± 1.0	0.570
Week 12	20.1 ± 0.9	21.8 ± 1.0	0.013
Intracellular fluid, L			
Baseline	24.2 ± 1.1	24.2 ± 1.2	Ref
Week 1	25.1 ± 1.1	24.1 ± 1.2	0.353
Week 12	23.4 ± 1.1	24.8 ± 1.2	0.254
Total body water, L			
Baseline	44.6 ± 1.9	44.3 ± 2.0	Ref
Week 1	44.7 ± 1.9	44.3 ± 2.0	0.985
Week 12	43.7 ± 2.0	46.3 ± 2.1	0.127
Total body water, %			
Baseline	50.4 ± 1.4	46.5 ± 1.5	Ref
Week 1	50.3 ± 1.4	46.4 ± 1.5	0.981
Week 12	48.9 ± 1.4	47.0 ± 1.6	0.165
Fat‐free mass, kg			
Baseline	60.8 ± 2.5	60.5 ± 2.7	Ref
Week 1	61.0 ± 2.5	60.6 ± 2.7	0.967
Week 12	60.1 ± 2.7	63.3 ± 2.9	0.176
Fat‐free mass, %			
Baseline	68.8 ± 2.0	62.6 ± 2.1	Ref
Week 1	68.7 ± 2.0	62.8 ± 2.1	0.843
Week 12	66.9 ± 2.0	63.5 ± 2.1	0.140
Fat mass, kg			
Baseline	29.0 ± 2.9	35.6 ± 3.1	Ref
Week 1	29.2 ± 2.9	35.8 ± 3.1	0.974
Week 12	32.5 ± 2.9	36.0 ± 3.1	0.239
Fat mass, %			
Baseline	31.0 ± 1.9	36.5 ± 2.1	Ref
Week 1	31.3 ± 1.9	36.6 ± 2.1	0.837
Week 12	33.2 ± 2.0	35.9 ± 2.1	0.133

*Note*: Values are in estimated means ± standard error of the mean except for estimated plasma volume, which is expressed in mean ± standard deviation. Estimated plasma volume was estimated using Strauss estimation (Liu et al., 2021; Lytvyn et al., 2017). 100 × (pre Hemoglobin (Hb)/post Hb) × ([100 − post Hematocrit (Ht)]/[100 − pre Ht]) −100. Statistical comparisons between placebo and ertugliflozin groups were performed using the Wilcoxon Rank Sum test except for estimated plasma volume. Estimated plasma volume was tested for equality of variances using the Welch–Satterthwaite equation.

### 3.6 Effects of ertugliflozin on neurohormonal and sympathetic activation

**FIGURE 5:**
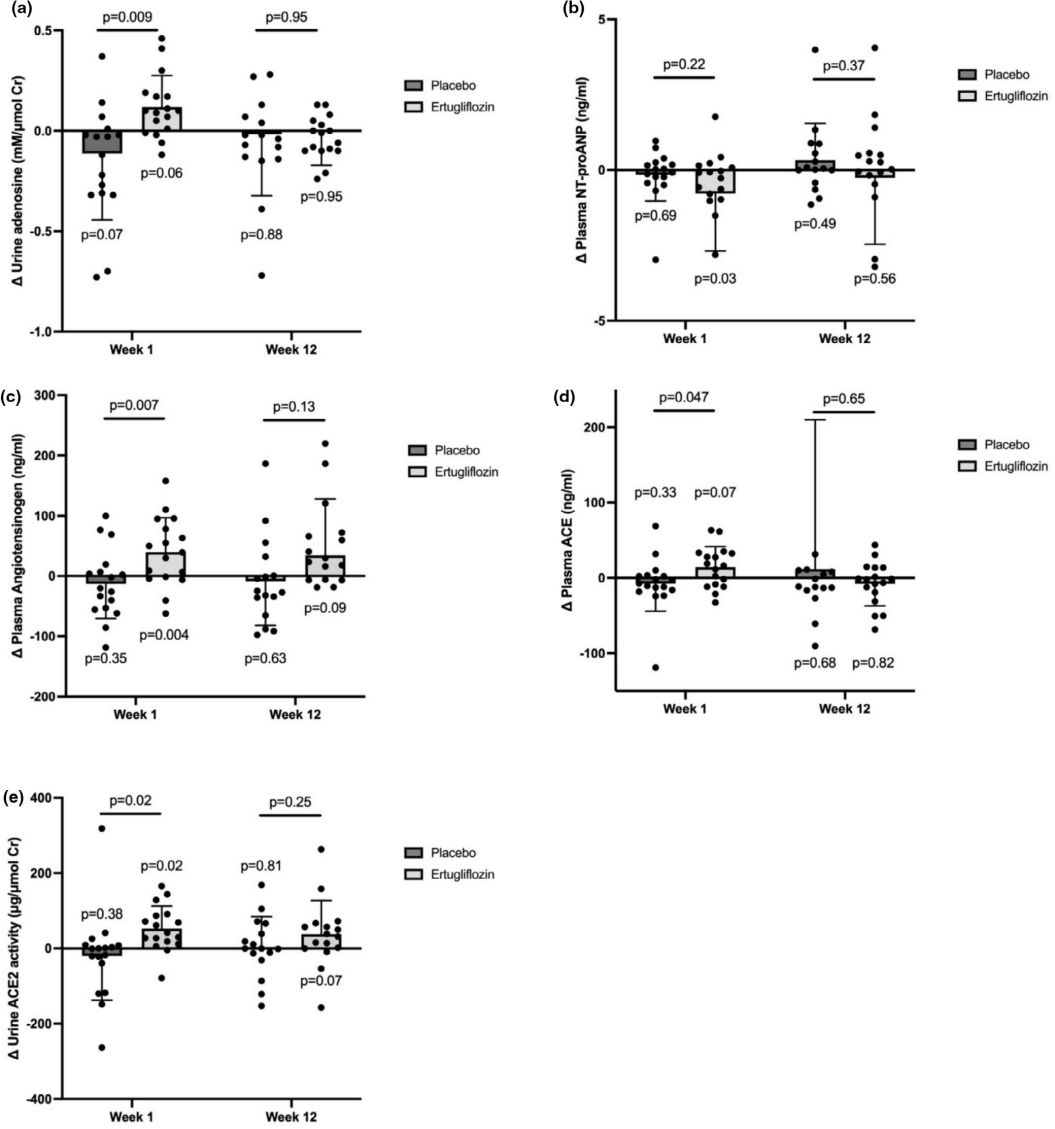
Changes in RAAS and natriuretic modulators after 1 week and 12 weeks of treatment. Changes in (a) urine adenosine, (b) plasma NT‐proANP, (c) plasma angiotensinogen, (d) plasma angiotensin‐converting enzyme (ACE), and (e) ACE‐2 activity during Week 1 and Week 12 of ertugliflozin treatment or placebo subtracted from baseline values.

Compared to placebo, ertugliflozin acutely increased plasma levels of angiotensinogen by 52.6 ± 19.7 pg/mL (*p* = 0.007) as well as ACE by 21.9 ± 11.1 ng/mL (*p* = 0.047) (Table 6 and Figure 5). NT‐proANP also acutely decreased with ertugliflozin (*p*_within group_ = 0.03), though these changes were not statistically different from the placebo group. BNP concentrations did not differ significantly between acute or chronic use of ertugliflozin compared to placebo. For urine markers, compared to placebo, ertugliflozin acutely increased urine adenosine (placebo‐adjusted increase of 0.23 ± 0.09 mM/μmol Cr, *p* = 0.009) and urine ACE2 activity (placebo‐adjusted increase of 0.07 ± 0.03 μg/μmol Cr, *p* = 0.02), although this was not sustained at 12 weeks (Figure 5). Ertugliflozin did not change urinary levels of other neurohormones or natriuretic modulators (Table 6). Moreover, vagal tone (RMSSD) and sympathetic activity (SDNN) measures, heart rate, and plasma norepinephrine and epinephrine concentrations were not significantly different with ertugliflozin use compared to placebo. In summary, ertugliflozin treatment demonstrated acute activation of neurohormonal pathways and significant changes in intraglomerular arteriolar regulation, without sympathetic activation.

**TABLE 6:** Neurohormonal and natriuretic modulators in response to ertugliflozin compared with placebo in participants with type 2 diabetes and heart failure.

	Ertugliflozin (*n* = 17), mean ± SE	Placebo (*n* = 17), mean ± SE	*p*‐Value placebo‐adjusted change from baseline
*Blood*			
Blood angiotensin II, pg/mL			
Baseline	90.2 ± 7.3	84.7 ± 7.3	Ref
Week 1	94.4 ± 7.3	89.0 ± 7.4	0.998
Week 12	89.2 ± 7.1	86.5 ± 7.3	0.802
Blood angiotensinogen, ng/mL			
Baseline	63.6 ± 18.0	84.4 ± 18.0	Ref
Week 1	103.3 ± 18.0	72.0 ± 18.0	0.007
Week 12	97.9 ± 19.3	74.8 ± 19.8	0.130
Renin, ng/L			
Baseline	82.1 ± 48.5	96.0 ± 48.5	Ref
Week 1	169.4 ± 48.5	100.7 ± 48.5	0.232
Week 12	117.6 ± 44.3	99.5 ± 45.4	0.617
Aldosterone, pmol/L			
Baseline	319.1 ± 69.0	397.2 ± 69.0	Ref
Week 1	354.8 ± 69.0	395.8 ± 69.0	0.205
Week 12	348.7 ± 67.0	400.2 ± 68.0	0.718
Blood ACE substrate, ng/mL			
Baseline	71.4 ± 28.1	97.4 ± 28.1	Ref
Week 1	85.7 ± 28.1	89.8 ± 28.1	0.047
Week 12	63.7 ± 30.7	111.4 ± 31.5	0.651
Blood ACE2, ng/mL			
Baseline	16.3 ± 7.7	5.9 ± 7.7	Ref
Week 1	13.7 ± 7.7	6.0 ± 7.7	0.217
Week 12	15.0 ± 8.1	5.0 ± 8.1	0.801
PRA, ng/mL/h			
Baseline	7.61 ± 1.80	7.49 ± 1.80	Ref
Week 1	8.74 ± 1.80	10.08 ± 1.80	0.412
Week 12	7.27 ± 1.88	7.96 ± 1.92	0.745
BNP, pg/mL			
Baseline	1478.9 ± 376.4	496.7 ± 376.4	Ref
Week 1	1080.9 ± 376.4	491.1 ± 376.4	0.178
Week 12	1311.1 ± 400.5	576.6 ± 405.3	0.327
Blood NT‐proANP, ng/mL			
Baseline	4.2 ± 0.8	2.0 ± 0.8	Ref
Week 1	3.4 ± 0.8	1.9 ± 0.8	0.224
Week 12	4.0 ± 0.8	2.4 ± 0.8	0.367
Norepinephrine, nM			
Baseline	2.5 ± 0.4	2.9 ± 0.3	Ref
Week 1	2.9 ± 0.3	2.8 ± 0.3	0.289
Week 12	2.6 ± 0.3	2.6 ± 0.3	0.618
Epinephrine, nM			
Baseline	0.16 ± 0.14	0.19 ± 0.13	Ref
Week 1	0.53 ± 0.13	0.22 ± 0.13	0.169
Week 12	0.11 ± 0.05	0.18 ± 0.05	0.620
*Urine*			
Urine angiotensin II, μg/μmol Cr			
Baseline	15.29 ± 3.72	22.32 ± 3.72	Ref
Week 1	13.44 ± 3.72	16.03 ± 3.72	0.512
Week 12	12.14 ± 3.74	16.99 ± 3.85	0.751
Urine angiotensinogen, mg/μmol Cr			
Baseline	2.53 ± 1.39	5.73 ± 139	Ref
Week 1	0.43 ± 1.39	2.77 ± 139	0.674
Week 12	0.41 ± 1.29	3.29 ± 1.33	0.896
Urine adenosine, mM/μmol Cr			
Baseline	0.25 ± 0.05	0.39 ± 0.05	Ref
Week 1	0.37 ± 0.05	0.28 ± 0.05	0.009
Week 12	0.25 ± 0.05	0.39 ± 0.05	0.946
Urine ACE substrate, μg/μmol Cr			
Baseline	0.019 ± 0.025	0.064 ± 0.025	Ref
Week 1	0.009 ± 0.025	0.074 ± 0.025	0.198
Week 12	0.028 ± 0.023	0.047 ± 0.024	0.268
Urine ACE activity, mg/μmol Cr			
Baseline	5.99 ± 3.64	4.87 ± 3.64	Ref
Week 1	14.0 ± 3.64	2.66 ± 3.64	0.086
Week 12	10.56 ± 4.87	11.8 ± 4.98	0.731
Urine ACE2 substrate, mg/μmol Cr			
Baseline	0.43 ± 0.17	0.87 ± 0.17	Ref
Week 1	0.33 ± 0.17	0.73 ± 0.17	0.868
Week 12	0.37 ± 0.20	0.90 ± 0.20	0.783
Urine ACE2 activity, μg/μmol Cr			
Baseline	0.053 ± 0.020	0.076 ± 0.020	Ref
Week 1	0.106 ± 0.020	0.056 ± 0.020	0.022
Week 12	0.091 ± 0.024	0.080 ± 0.024	0.255
Urine NT‐proANP, mg/μmol Cr			
Baseline	0.28 ± 0.08	0.40 ± 0.08	Ref
Week 1	0.36 ± 0.08	0.32 ± 0.08	0.188
Week 12	0.31 ± 0.07	0.29 ± 0.08	0.219

*Note*: Values are in estimated means ± standard error of the mean. Urine markers are normalized to urine creatinine. Statistical comparisons between placebo and ertugliflozin groups were performed using the Wilcoxon Rank Sum test.

Abbreviations: ACE, angiotensin‐converting enzyme; ANP; atrial natriuretic peptide; BNP, brain natriuretic peptide; PRA, plasma renin activity; SE, standard error of the mean.

### 3.7 Effect of ertugliflozin on metabolic characteristics, plasma, and urine biochemistry

**FIGURE 6:**
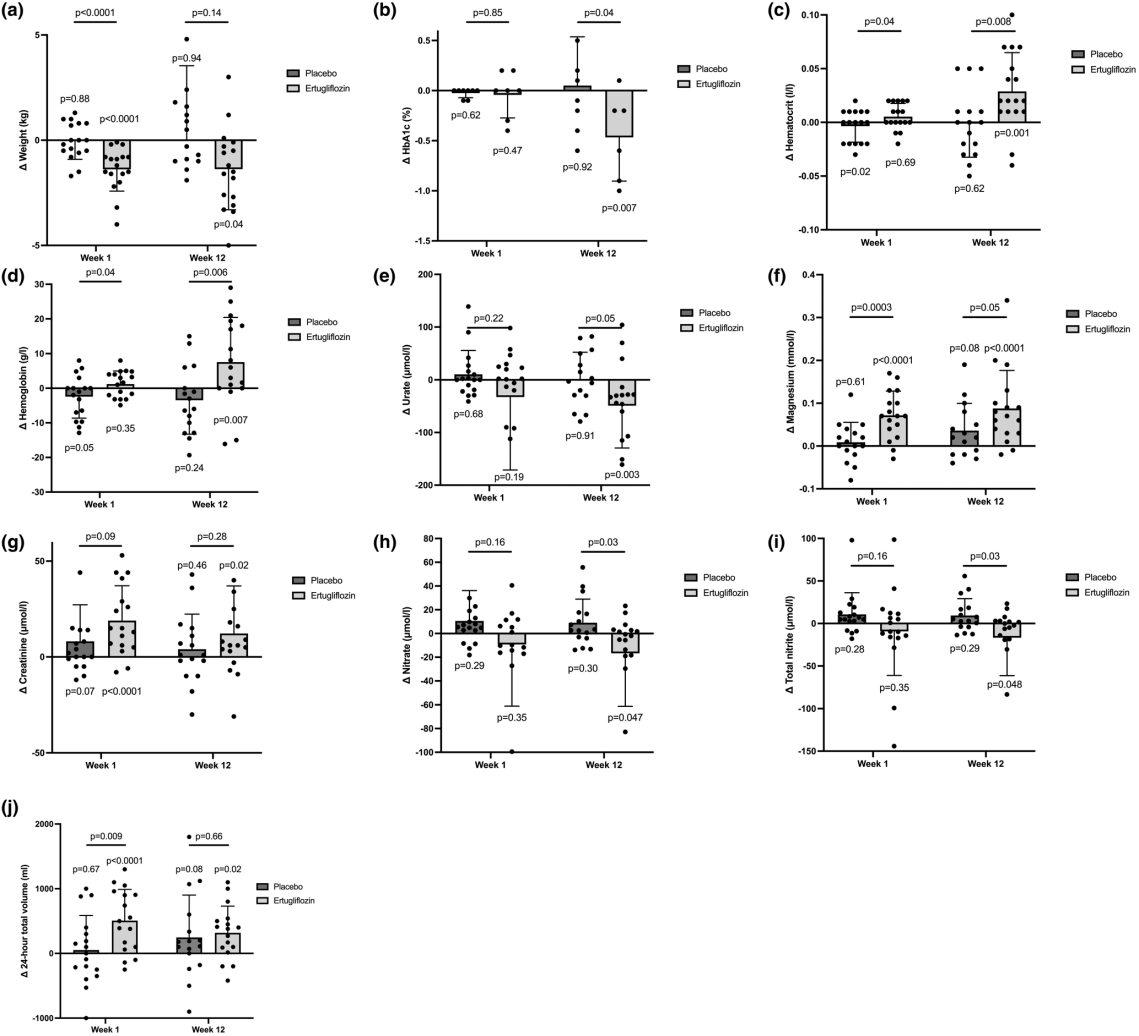
Changes in metabolic, plasma, and urine biochemistry after 1 week and 12 weeks of treatment. Changes in (a) weight, (b) HbA1c, (c) hematocrit, (d) hemoglobin, (e) urate, (f) magnesium, (g) creatinine, (h) nitrate, (i) total nitrite, and (j) 24‐h volume during Week 1 and Week 12 of ertugliflozin or placebo subtracted from baseline values.

Compared to placebo, ertugliflozin treatment decreased weight by 1.35 ± 0.33 kg (*p* < 0.0001) at 1 week, which was not sustained with 12 weeks of treatment compared to placebo (Figure 6a). Ertugliflozin treatment for 12 weeks did not significantly change waist circumference or fasting plasma glucose levels (Table 7). Compared to placebo, the addition of ertugliflozin decreased HbA1c by 0.53 ± 0.25% (*p* = 0.04) at 12 weeks (Figure 6b). Ertugliflozin increased hematocrit compared with placebo at 1 week (0.013 ± 0.006 g/L, *p* = 0.04) and 12 weeks (0.035 ± 0.013 g/L, *p* = 0.008), with corresponding placebo‐adjusted increases in hemoglobin at 1 and 12 weeks (Figure 6c,d). Levels of magnesium increased significantly after 1 week of ertugliflozin (*p*_within group_ = 0.0003) and remained augmented after 12 weeks of ertugliflozin (*p*_within group_ <0.0001), but the sustained changes were not significantly different compared with placebo.

**TABLE 7:** Metabolic characteristics and plasma and urine biochemistry responses to ertugliflozin compared with placebo in participants with type 2 diabetes and heart failure.

	Ertugliflozin (*n* = 17), mean ± SE	Placebo (*n* = 17), mean ± SE	*p*‐Value placebo‐adjusted change from baseline
*Metabolic characteristics*			
Weight, kg			
Baseline	91.85 ± 4.29	96.89 ± 4.29	Ref
Week 1	90.47 ± 4.29	96.85 ± 4.29	<0.0001
Week 12	90.47 ± 4.40	96.94 ± 4.40	0.144
BMI, kg/m^2^			
Baseline	30.0 ± 1.4	31.8 ± 1.5	Ref
Week 1	29.8 ± 1.4	31.9 ± 1.5	0.556
Week 12	30.2 ± 1.4	32.6 ± 1.5	0.499
Waist circumference, cm			
Baseline	109.5 ± 3.4	118.0 ± 3.4	Ref
Week 1	109.4 ± 3.4	118.0 ± 3.4	0.929
Week 12	109.4 ± 3.3	117. 7 ± 3.3	0.924
Fasting plasma glucose, mmol/L			
Baseline	8.6 ± 0.6	7.3 ± 0.6	Ref
Week 1	8.7 ± 0.6	6.6 ± 0.6	0.390
Week 12	7.7 ± 0.6	7.5 ± 0.6	0.325
HbA1c, %			
Baseline	8.2 ± 0.3	7.6 ± 0.3	Ref
Week 1	8.2 ± 0.3	7.6 ± 0.3	0.851
Week 12	7.7 ± 0.3	7.6 ± 0.3	0.036
*Plasma biochemistry*			
Hematocrit, L/L			
Baseline	0.37 ± 0.01	0.39 ± 0.01	Ref
Week 1	0.38 ± 0.01	0.38 ± 0.01	0.044
Week 12	0.41 ± 0.02	0.40 ± 0.02	0.008
Hemoglobin, g/L			
Baseline	126.4 ± 4.5	131.1 ± 4.5	Ref
Week 1	127.4 ± 4.5	128.6 ± 4.5	0.039
Week 12	133.8 ± 5.0	127.7 ± 5.0	0.006
Plasma albumin, g/L			
Baseline	38.6 ± 0.80	38.8 ± 0.80	Ref
Week 1	39.5 ± 0.80	39.3 ± 0.80	0.481
Week 12	38.1 ± 0.88	39.1 ± 0.91	0.489
Blood urea, mmol/mol			
Baseline	22.1 ± 6.1	8.5 ± 6.1	Ref
Week 1	10.7 ± 6.1	9.0 ± 6.1	0.320
Week 12	10.2 ± 6.1	8.6 ± 6.3	0.326
Blood urate, μmol/L			
Baseline	405.8 ± 29.1	451.8 ± 29.1	Ref
Week 1	373.2 ± 29.1	461.8 ± 29.1	0.223
Week 12	356.6 ± 25.9	449.3 ± 26.6	0.051
AST, U/L			
Baseline	27.9 ± 2.2	21.5 ± 2.2	Ref
Week 1	24.7 ± 2.2	20.8 ± 2.2	0.197
Week 12	25.0 ± 2.5	24.4 ± 2.6	0.117
ALT, U/L			
Baseline	27.6 ± 2.7	20.3 ± 2.7	Ref
Week 1	24.2 ± 2.7	19.4 ± 2.7	0.102
Week 12	23.2 ± 2.6	19.3 ± 2.7	0.207
ALP, U/L			
Baseline	82.5 ± 7.5	85.1 ± 7.1	Ref
Week 1	77.2 ± 7.5	80.7 ± 7.2	0.879
Week 12	73.8 ± 7.1	80.9 ± 7.0	0.688
LDL, mmol/L			
Baseline	1.86 ± 0.17	1.72 ± 0.17	Ref
Week 1	1.92 ± 0.17	1.53 ± 0.17	0.018
Week 12	1.59 ± 0.17	1.76 ± 0.17	0.192
HDL, mmol/L			
Baseline	1.06 ± 0.08	1.00 ± 0.08	Ref
Week 1	1.01 ± 0.09	1.04 ± 0.08	0.309
Week 12	1.01 ± 0.07	0.97 ± 0.07	0.843
Triglyceride, mmol/L			
Baseline	1.56 ± 0.24	1.78 ± 0.24	Ref
Week 1	1.89 ± 0.24	1.70 ± 0.24	0.013
Week 12	2.19 ± 0.43	1.78 ± 0.44	0.341
Plasma magnesium, mmol/L			
Baseline	0.78 ± 0.02	0.72 ± 0.02	Ref
Week 1	0.85 ± 0.02	0.72 ± 0.02	0.0003
Week 12	0.86 ± 0.02	0.76 ± 0.02	0.053
Plasma creatinine, μmol/L			
Baseline	118.5 ± 10.3	104.8 ± 10.3	Ref
Week 1	137.4 ± 10.3	112.9 ± 10.3	0.090
Week 12	130.7 ± 10.1	108.9 ± 10.2	0.281
Blood cGMP, pmol/mL			
Baseline	56.2 ± 14.6	48.9 ± 14.6	Ref
Week 1	55.1 ± 14.6	56.7 ± 14.6	0.251
Week 12	57.5 ± 14.6	56.0 ± 14.6	0.389
Blood nitrate, μmol/L			
Baseline	60.6 ± 12.6	34.7 ± 12.6	Ref
Week 1	51.4 ± 12.6	45.2 ± 12.6	0.157
Week 12	43.9 ± 12.1	43.6 ± 12.3	0.034
Blood total nitrite, μmol/L			
Baseline	72.3 ± 12.6	46.5 ± 12.6	Ref
Week 1	63.1 ± 12.6	57.1 ± 12.6	0.157
Week 12	55.6 ± 12.1	55.7 ± 12.3	0.032
Blood endogenous nitrite, μmol/L			
Baseline	11.7 ± 0.1	11.8 ± 0.1	Ref
Week 1	11.7 ± 0.1	11.8 ± 0.1	0.761
Week 12	11.6 ± 0.1	12.0 ± 0.1	0.139
Blood PGF2α, pg/mL			
Baseline	341.5 ± 30.5	286.8 ± 30.5	Ref
Week 1	363.5 ± 30.5	319.9 ± 30.5	0.807
Week 12	361.3 ± 30.6	322.6 ± 31.2	0.688
*Urine biochemistry*			
24‐h urine total volume, mL			
Baseline	1952 ± 186	2014 ± 186	Ref
Week 1	2459 ± 186	2066 ± 186	0.009
Week 12	2270 ± 194	2249 ± 196	0.660
24‐h urea excretion, mmol/day			
Baseline	360 ± 37	291 ± 37	Ref
Week 1	353 ± 37	314 ± 37	0.390
Week 12	326 ± 33	282 ± 36	0.514
24‐h potassium excretion, mmol/day			
Baseline	71 ± 7	59 ± 7	Ref
Week 1	73 ± 7	70 ± 7	0.295
Week 12	73 ± 7	72 ± 7	0.100
24‐h sodium excretion, mmol/day			
Baseline	156.1 ± 20.6	157.2 ± 20.6	Ref
Week 1	197.3 ± 20.3	150.8 ± 20.6	0.032
Week 12	155.5 ± 17.9	177.9 ± 18.9	0.512
24‐h protein excretion, g/day			
Baseline	0.352 ± 0.123	0.405 ± 0.123	Ref
Week 1	0.291 ± 0.122	0.406 ± 0.123	0.486
Week 12	0.454 ± 0.166	0.568 ± 0.172	0.698
24‐h UACR, mg/mmol			
Baseline	44.0 ± 19.8	23.2 ± 19.8	Ref
Week 1	36.6 ± 19.8	20.5 ± 19.9	0.473
Week 12	36.4 ± 15.3	41.1 ± 16.0	0.809
Urine 8‐isoprostane, ng/μmol Cr			
Baseline	0.065 ± 0.013	0.074 ± 0.013	Ref
Week 1	0.083 ± 0.013	0.051 ± 0.014	0.070
Week 12	0.072 ± 0.024	0.101 ± 0.025	0.572
Urine 8‐OHdG, μg/μmol Cr			
Baseline	0.035 ± 0.012	0.057 ± 0.011	Ref
Week 1	0.060 ± 0.011	0.039 ± 0.011	0.029
Week 12	0.032 ± 0.009	0.046 ± 0.010	0.622
Urine cGMP, pmol/μmol Cr			
Baseline	0.014 ± 0.006	0.028 ± 0.006	Ref
Week 1	0.016 ± 0.006	0.016 ± 0.006	0.141
Week 12	0.013 ± 0.007	0.019 ± 0.007	0.486
Urine nitrate, μmol/μmol Cr			
Baseline	0.070 ± 0.020	0.079 ± 0.020	Ref
Week 1	0.066 ± 0.020	0.048 ± 0.020	0.325
Week 12	0.066 ± 0.024	0.076 ± 0.024	0.958
Urine total nitrite, μmol/μmol Cr			
Baseline	0.075 ± 0.021	0.088 ± 0.021	Ref
Week 1	0.072 ± 0.021	0.053 ± 0.021	0.287
Week 12	0.071 ± 0.025	0.083 ± 0.026	1.000
Urine endogenous nitrite, μmol/μmol Cr			
Baseline	0.005 ± 0.002	0.009 ± 0.002	Ref
Week 1	0.006 ± 0.002	0.006 ± 0.002	0.069
Week 12	0.005 ± 0.002	0.007 ± 0.002	0.486
Urine PGF2α, ng/μmol Cr			
Baseline	0.036 ± 0.009	0.051 ± 0.009	Ref
Week 1	0.041 ± 0.009	0.032 ± 0.009	0.045
Week 12	0.050 ± 0.014	0.049 ± 0.015	0.477
Urine sodium, mmol/μmol Cr			
Baseline	0.016 ± 0.005	0.024 ± 0.005	Ref
Week 1	0.017 ± 0.005	0.015 ± 0.005	0.186
Week 12	0.011 ± 0.005	0.019 ± 0.005	0.958
Urine urea, mmol/μmol Cr			
Baseline	204.1 ± 26.2	181.0 ± 26.2	Ref
Week 1	177.8 ± 26.2	207.8 ± 26.2	0.205
Week 12	202.1 ± 27.3	200.3 ± 28.0	0.606
UACR, mg/mmol Cr			
Baseline	16.3 ± 7.0	27.8 ± 7.0	Ref
Week 1	10.5 ± 7.0	18.0 ± 7.0	0.695
Week 12	24.7 ± 11.2	22.9 ± 11.5	0.869
Urine protein, g/μmol Cr			
Baseline	0.4 ± 1.6	0.4 ± 1.6	Ref
Week 1	3.3 ± 1.6	0.3 ± 1.6	0.317
Week 12	0.3 ± 0.1	0.5 ± 0.1	0.399
Urine potassium, mmol/μmol Cr			
Baseline	39.7 ± 5.1	35.6 ± 5.1	Ref
Week 1	33.7 ± 5.1	44.7 ± 5.1	0.042
Week 12	44.2 ± 5.5	38.2 ± 5.6	0.811

*Note*: Values are in estimated means ± standard error of the mean. Waist circumference was measured at screening. Urine markers are normalized to urine creatinine. Statistical comparisons between placebo and ertugliflozin groups were performed using the Wilcoxon Rank Sum test.

Abbreviations: ALP; alkaline phosphatase; ALT, alanine transaminase; AST, aspartate transaminase; cGMP, cyclic guanosine monophosphate; HbA1c, hemoglobin A1c; HDL, high‐density lipoprotein; LDL, low‐density lipoprotein; PGF2α, prostaglandin F2α; SE, standard error of the mean; UACR, urine albumin‐to‐creatinine ratio.

Compared to placebo, ertugliflozin significantly decreased plasma nitric oxide levels as observed by the decrease in nitrate by 25.7 ± 12.1 μmol/L (*p* = 0.03) and total nitrite by 26.0 ± 12.1 μmol/L (*p* = 0.03) at 12 weeks, without changes to endogenous nitrite levels (Figure 6h,i). No other sustained changes in oxidative stress markers were observed in plasma and urine (Table 7). Plasma uric acid levels decreased with ertugliflozin by 49.1 μmol/L (*p*_within group_ = 0.003), although these changes were not statistically different from placebo. There was no change in total urea and potassium excretion between or within treatment groups (Tables 7 and S4). No significant changes to other plasma or urine biochemistry markers were observed. In conclusion, ertugliflozin led to a significant initial weight loss and a reduction in HbA1c, suggesting early metabolic benefits that could be advantageous for patients with T2D. Additionally, changes in hematocrit, hemoglobin, and magnesium levels may reflect favorable mediation of these metabolic and biochemical markers.

### 3.8 Adverse events

Three participants, all randomized to ertugliflozin treatment, experienced six serious adverse events (SAEs). One participant had four SAEs—three HF hospitalizations and one cerebrovascular accident. The second participant was hospitalized with emesis, and the third participant had a myocardial infarction. One participant randomized to the placebo group underwent early end of treatment due to ongoing emesis. There were no episodes of ketoacidosis or major hypoglycemic events with placebo or ertugliflozin treatment.

## 4 DISCUSSION

SGLT2 inhibitors are an integral part of guideline‐directed medical therapy in HF patients with either reduced or preserved ejection fraction (Damman et al., 2020; McDonagh et al., 2023). The current mechanistic study evaluated the cardio‐kidney‐metabolic physiological effects of ertugliflozin in participants with T2D and HF. Ertugliflozin had no impact on the primary outcome measure of proximal sodium reabsorption using morning spot urine samples collected during clamped euglycemia. However, 24‐h urine sodium excretion and urine volume did increase acutely at 1 week in response to ertugliflozin, along with a decline in left ventricular stroke volume and higher levels of circulating and urine RAAS components—with no changes in these parameters at Week 12. Moreover, at Week 12, extracellular fluid, estimated plasma volume, and blood pressure were lower with ertugliflozin versus placebo. The impact of ertugliflozin on other kidney and systemic hemodynamics and neurohormones remained generally unchanged versus placebo. Taken together, these results emphasize the predominantly acute effect of SGLT2 inhibition on cardiorenal physiology, which together may lead to persistent reductions in markers of volume overload, thereby contributing to a lower risk of HF progression.

The primary outcome of this trial was the change in FELi, representing proximal tubular sodium excretion. Neither an acute nor a chronic increase in proximal tubular sodium excretion was observed in this cohort of participants, the majority of whom were on baseline loop diuretic therapy. Fractional excretion of sodium and distal tubular sodium reabsorption (FE_Li_ − FE_Na_) with ertugliflozin also remained unchanged. We hypothesized that SGLT2 inhibition would acutely increase proximal tubular natriuresis with parallel increases in total natriuresis. However, over longer periods of time beyond several days, counterregulatory adaptation to tubular sodium handling, including reductions in proximal sodium excretion and increases in distal sodium reabsorption, may attenuate total natriuresis (Rao et al., 2024; Zannad et al., 2022). While changes in proximal sodium absorption were not observed in this cohort, an acute increase in urine volume of approximately 500 mL and total sodium excretion was observed. Increases in urine volume and total sodium excretion in the absence of changes in fractional lithium and sodium excretion may have several explanations and could simply reflect high anticipated levels of variability in morning sodium excretion in patients taking loop diuretics. Alternatively, passive osmotic diuresis with glucosuria may have been a driver of 24‐h natriuresis in this study. This, however, runs counter to prior studies in HF participants where no correlation was observed between metrics of glucosuria and natriuresis (Griffin et al., 2020; Rao et al., 2024). Neutral effects on various natriuresis measures by 12 weeks are consistent with findings in other studies demonstrating that counterregulatory adaptations to tubular sodium handling may offset the acute and transient diuretic effects of SGLT2 inhibition in chronic HF patients (Packer et al., 2023).

Despite neutral effects on measures of natriuresis at 12 weeks, ertugliflozin did lower extracellular fluid measured via bioimpedance spectroscopy and estimated plasma volume. Importantly, these reductions in volume markers were sustained at 12 weeks. The modest effects observed in our study are consistent with existing literature. The EMPIRE‐HF trial examined the change in extracellular volume in HF patients randomized to empagliflozin versus placebo for 12 weeks (Jensen et al., 2021) and showed that empagliflozin reduced estimated extracellular volume compared with placebo by a modest 0.12 L. Other studies also examined changes in extracellular volume with SGLT2 inhibition using bioimpedance spectroscopy and demonstrated reductions ranging from 0.5 to 1 L, which tended to attenuate over time. The consistent effects of SGLT2 inhibitors on plasma volume, extracellular fluid, and urine output across various HF populations, including acute decompensated HF and stable HF, have been previously discussed (Nardone et al., 2024). Notably, many of these volume mechanisms are not limited to HF patients but also extend to nondiabetic, non‐HF patients, as well as to patients with type 1 diabetes (Cherney et al., 2014, 2020; Scholtes et al., 2022). In contrast with this previous work, plasma volume in the current study was estimated using the Strauss equation, which evaluates changes in hematocrit relative to hemoglobin, both of which increased with ertugliflozin over time (Strauss et al., 1951). Analyses of SGLT2 inhibitor outcome trials have identified changes in hematocrit to be most closely associated with improvements in cardiovascular and kidney outcomes, consistent with plasma volume contraction and cardiorenal protection (Inzucchi et al., 2018; Li, Neal, et al., 2020; Li, Woodward, et al., 2020; Segar et al., 2022). One major limitation of using the Strauss equation to estimate plasma volume in the context of SGLT2 inhibitors is the potential effects of this class of drugs on erythropoietin and reticulocytosis (Lawler et al., 2021; Packer, 2023). Regardless, changes in estimated plasma volume, bioimpedance‐measured extracellular fluid, blood pressure, stroke volume (which can be reduced due to a decrease in cardiac preload), and body weight observed in this study were all concordant and suggestive of systemic volume reduction, although these changes were not accompanied by evidence of sympathetic activation. Also consistent with systemic volume contraction was the demonstration of acute RAAS activation that tended to attenuate over time—effects that have been mirrored in previous work involving patients with CKD (Lytvyn et al., 2022).

Ertugliflozin was associated with acute and sustained reductions in supine MAP compared to placebo as well as acute reductions in seated DBP. There are likely multiple mechanisms behind the observed blood pressure lowering. One is a decline in systemic intravascular volume, as reflected by reductions in estimated plasma volume and extracellular fluid, leading to blood pressure reductions. An acute reduction in total peripheral resistance with ertugliflozin, which has also been reported in other cohorts using other SGLT2 inhibitors, may have also contributed to blood pressure lowering, although the change in peripheral resistance was not different compared to placebo and therefore was unlikely to have played a major role.

In the current trial, ertugliflozin was associated with an acute within‐group dip in measured GFR in a population with T2D, 80% background use of renin–angiotensin blockade, and 97% background use of diuretic therapy. SGLT2 inhibition is known to result in an acute and reversible 4–6 mL/min/1.73 m^2^ dip in GFR, although the exact hemodynamic mechanism that is responsible remains controversial. It has been suggested that changes in TGF may account for both the short‐term “dip” in GFR and the long‐term benefits attenuating GFR decline with SGLT2 inhibition. According to this hypothesis, SGLT2 inhibition decreases sodium and glucose reabsorption in the proximal nephron, leading to distal sodium delivery to the macula densa. Sodium from the tubular lumen enters the macula densa through Na^+^/K^+^/2Cl^−^ cotransporters and stimulates the local generation of adenosine triphosphate and/or adenosine (Tonneijck et al., 2017). By increasing distal sodium delivery, SGLT2 inhibitors are hypothesized to restore TGF and have been demonstrated to reduce calculated intraglomerular pressure (Cherney et al., 2014). However, this mechanism has limitations. As the TGF mechanism adapts over time, the benefits of SGLT2 inhibitors are also observed in individuals without diabetes (Cherney et al., 2020; Group E‐KC, 2023), where the effect on proximal sodium reabsorption is less pronounced (Cherney et al., 2018; Ferrannini et al., 2017). Moreover, less clear is whether reductions in intraglomerular pressure are mediated via preglomerular arteriolar constriction or postglomerular arteriolar vasodilation, with studies demonstrating that the net effect on arteriolar tone may differ between T1D and T2D populations and between people with or without glomerular hyperfiltration (Liu et al., 2021). In our cohort of participants with T2D and HF, SGLT2 inhibition resulted in a decrease in GFR without significant changes to RBF or RVR, making it challenging to discern whether postglomerular arteriolar resistance or preglomerular arteriolar resistance was mainly involved. Nevertheless, the absence of effects on ERPF and RVR makes it less likely that preglomerular vasoconstriction is the primary hemodynamic mechanism mediating eGFR decline. Another possibility is that the amelioration of volume overload due to osmotic diuresis could reduce blood volume and pressure, further contributing to a decrease in GFR (Lambers Heerspink et al., 2013). Irrespective of the responsible mechanisms, increases observed with ertugliflozin in urine adenosine and decreases in nitric oxide, a neurohormonal factor that counters the former's preglomerular vasoconstriction, suggest activation of TGF mechanisms, at least within 1 week of initiation of therapy (Cherney et al., 2012; Rajasekeran et al., 2017; Thorup & Persson, 1994; Turkstra et al., 1998).

There are limitations to the present analysis. Due to the increasing use of SGLT2 inhibition for the clinical indication of HF and challenges posed by the COVID‐19 pandemic, we were unable to meet the participant recruitment target for this trial. Therefore, our study was potentially underpowered to detect a 40% difference between ertugliflozin and placebo groups in the primary outcome of FE_Li_. Finally, this was a multicenter mechanistic study involving stable participants with HF and T2D. Results of HF and kidney outcome trials now demonstrate clinical benefit irrespective of diabetes status—it remains to be seen whether the results of the present study are also applicable to the participants with HF but without T2D.

## 5 PERSPECTIVES AND SIGNIFICANCE

SGLT2 inhibition is an integral part of guideline‐directed medical therapy in HF patients with either reduced or preserved ejection fraction. However, the mechanisms underlying the cardio‐kidney‐metabolic effect of SGLT2 inhibition in patients with T2D and HF have not been completely elucidated. In the current study, we found that ertugliflozin did not reduce proximal or distal sodium excretion but acutely increased 24‐h natriuresis and urine volume at 1 week, which was attenuated at 12 weeks. These findings likely represent counterregulatory adaptation to tubular sodium handling, including reductions in proximal sodium excretion and increases in distal sodium reabsorption, and are consistent with other studies reporting neutral effects on natriuresis measures by 12 weeks. In the context of a plasma volume decline at 12 weeks, our findings suggest that SGLT2 inhibition shifts systemic volume toward a state of euvolemia, thereby lowering the risk of worsening HF, although it is unclear to what extent, if any, these benefits are mediated by the diuretic effects of SGLT2 inhibitors.

## AUTHOR CONTRIBUTIONS

YL, RAS, EMB, VSS, LK, DHR, HJLH, and DZIC contributed to the initial draft and review concept, design, and manuscript preparation. All authors have contributed to the writing of the manuscript—provided critical edits, reviewed, and approved the final manuscript. No writing assistance was received by the authors. DZIC is the guarantor of this work.

## FUNDING INFORMATION

The study was an investigator‐initiated study, entirely planned and conducted under the scientific supervision of DHR, HJLH, and DZIC. This study was supported in part by a research grant from the Investigator‐Initiated Studies Program of Merck Canada Inc. The opinions expressed in this paper are those of the authors and do not necessarily represent those of Merck Canada Inc. The funders had no role in the study design, analysis or interpretation of the data, or drafting of the manuscript. The funders had no role in the decision to submit this manuscript for publication.

## DISCLOSURES

DZIC has received honoraria from Boehringer Ingelheim–Lilly, Merck, AstraZeneca, Sanofi, Mitsubishi–Tanabe, Abbvie, Janssen, Bayer, Prometic, BMS, Maze, Gilead, CSL–Behring, Otsuka, Novartis, Youngene, Lexicon, and Novo–Nordisk and has received operational funding for clinical trials from Boehringer Ingelheim–Lilly, Merck, Janssen, Sanofi, AstraZeneca, CSL–Behring, and Novo–Nordisk. VSS is supported by the Department of Medicine Eliot Phillipson Clinician Scientist Training Program and a Banting and Best Diabetes Centre Postdoctoral fellowship at the University of Toronto. VSS has received conference and travel support from Merck Canada. YL, LK, LEL, and EMB have no conflicts of interest to disclose. DHvR has acted as a consultant for and received honoraria from Boehringer Ingelheim–Lilly Diabetes Alliance, Merck, Sanofi, and AstraZeneca and has received research operating funds from Boehringer Ingelheim–Lilly Diabetes Alliance, AstraZeneca, and Merck (all honoraria transferred to employer Amsterdam UMC). The employer of AAV received consultancy fees and/or research support from Adrenomed, Anacardio, AstraZeneca, Bayer AG, BMS, Boehringer Ingelheim, Corteria, Eli Lilly, Merck, Moderna, Novartis, Novo Nordisk, Roche Diagnostics, and SalubrisBio.

## ETHICS STATEMENT

The local Research Ethics Board at the University Health Network (Toronto, Canada), University Medical Center Groningen (Groningen, Netherlands), and Amsterdam University Medical Centre (Amsterdam, Netherlands) approved the protocol, and all subjects gave informed consent prior to the start of procedures. The study was conducted according to the International Conference on Harmonization on Good Clinical Practice.

## Supporting information


Figure S1.

## Data Availability

Source data for this study are not publicly available due to privacy or ethical restrictions. The source data are available to verified researchers upon request by contacting the corresponding author.
