# Supplementary material for: Mechanistic evaluation of ertugliflozin in patients with type 2 diabetes and heart failure
Source: Physiol Rep. 2025 Apr 10;13(7):e70275. doi: 10.14814/phy2.70275 (PMC11983784; doi:10.14814/phy2.70275)
Supplement: Supplementary file 1 — Figure S1. [file PHY2-13-e70275-s001.docx]

**SUPPLEMENTAL MATERIALS**


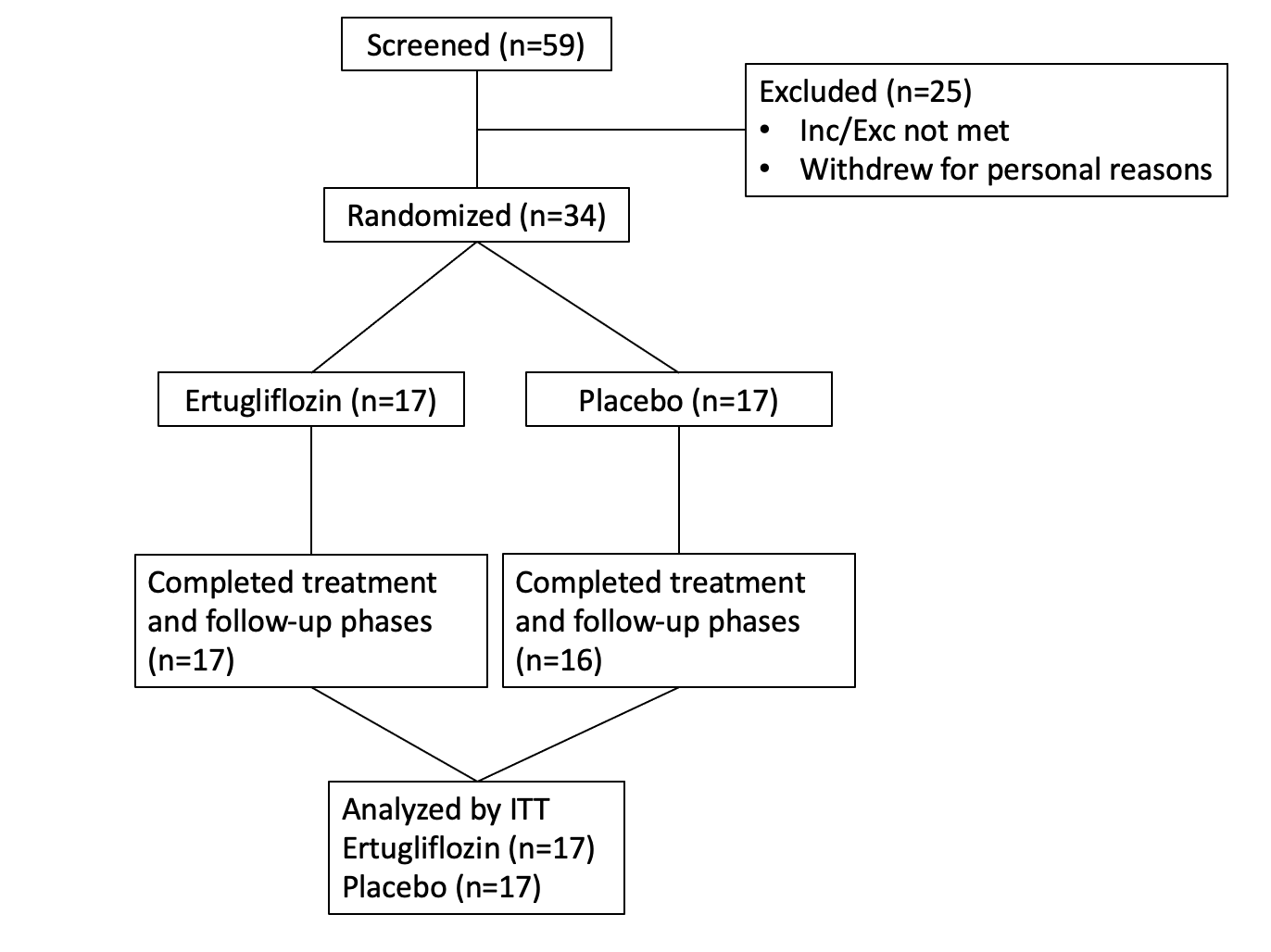


**Supplemental Figure S1.** Flow diagram for study participants.

**Supplemental Figure S2.** Study design for ERADICATE trial.

**
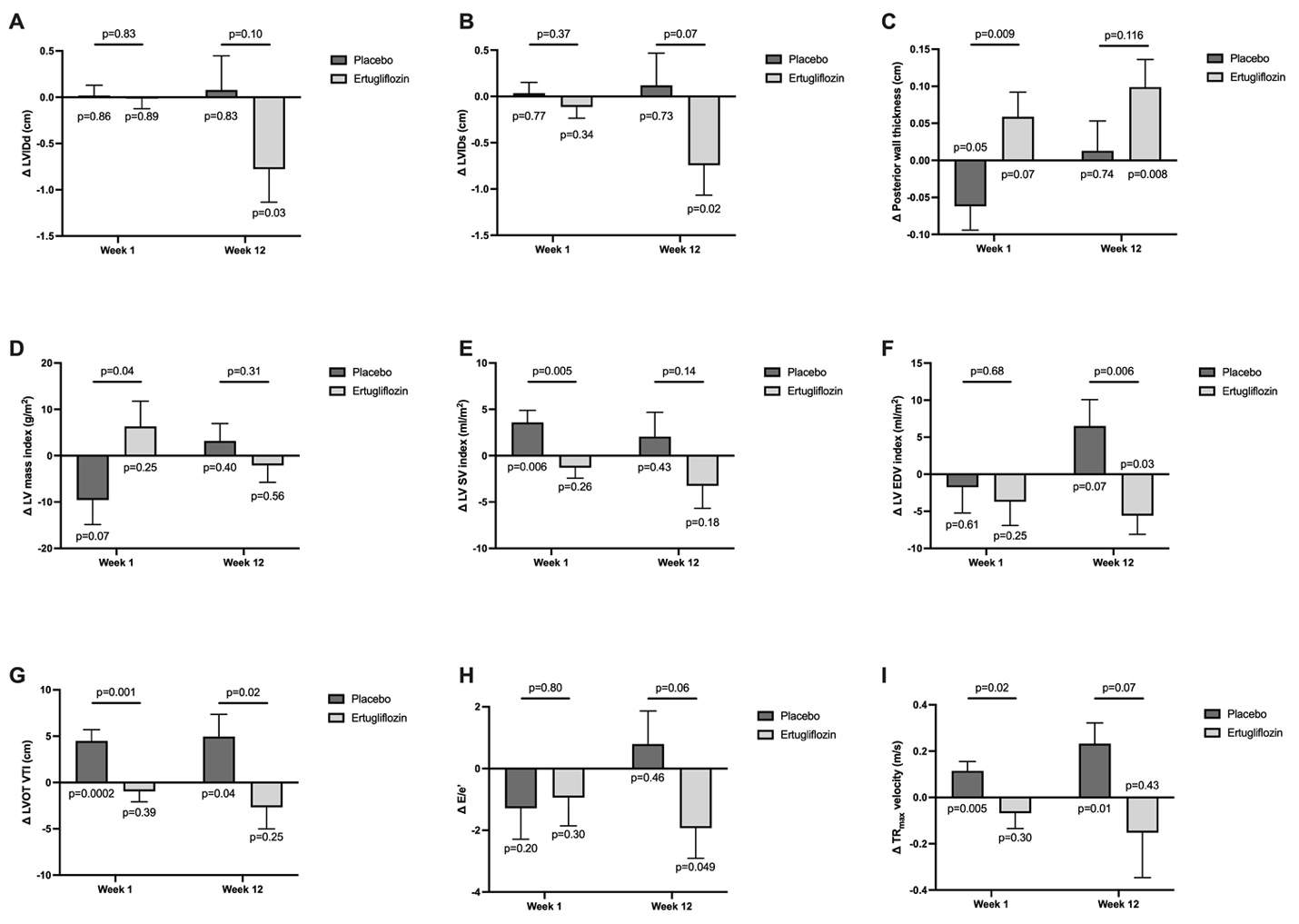
**

**Supplemental Figure S3. Changes in echocardiographic parameters after 1 week and 12 weeks of treatment.** Changes in A) left ventricular internal diameter end diastole (LVIDd), B) left ventricular internal diameter end systole (LVIDs), C) posterior wall thickness, D) indexed left ventricular mass (LV mass index), E) indexed left ventricular stroke volume (LV SV index), F) indexed left ventricular end-diastolic volume (LV EDV index), G) left ventricular outflow tract velocity time integral (LVOT VTI), H) E/e’, and I) maximal tricuspid regurgitation velocity (TR_max_ velocity) during week 1 and week 12 of ertugliflozin treatment or placebo subtracted from baseline values.

**Supplemental Table S1. Detailed inclusion and exclusion criteria**

| Inclusion criteria | |
| --- | --- |
| 1. | Male or female subjects diagnosed with T2D ≥12 months prior to informed consent |
| 2. | eGFR ≥30 ml/min/1.73m^2^ |
| 3. | Age >18 years |
| 4. | HbA1c 6.5%-10.5% |
| 5. | Body Mass Index (BMI) 18.5-45.0 kg/m^2^ |
| 6. | Blood pressure ≤160/110 and ≥90/60 at screening |
| 7. | Heart failure with New York Heart Association (NYHA) class 2-3 symptoms and ejection fraction ≥20% |
| 8. | Stable dose of maximally tolerated ACE inhibitor, angiotensin receptor blocker or renin inhibitor for at least 30 days |
| 9. | Stable diuretic dose for at least 30 days at the time of baseline physiological assessment |
| 10. | BNP levels at baseline ≥100 pg/ml (no atrial fibrillation), ≥200 pg/ml if in atrial fibrillation |

| Exclusion criteria | |
| --- | --- |
| 1. | Type 1 Diabetes |
| 2. | Leukocyte and/or nitrite positive urinalysis that is untreated |
| 3. | Severe hypoglycaemia within 2 months prior to screening |
| 4. | History of brittle diabetes or hypoglycaemia unawareness based on investigator judgement |
| 5. | Unstable coronary artery disease with acute coronary syndrome, percutaneous intervention or bypass surgery within 3 months |
| 6. | Clinically significant valvular disease |
| 7. | Congestive heart failure secondary to an infiltrative cardiomyopathic process (for example amyloid) or pericardial constriction |
| 8. | Uncontrolled systemic hypertension (systolic blood pressure >160 mmHg and/or diastolic blood pressure >110) or systemic hypotension (systolic blood pressure < 90/60 mmHg) |
| 9. | Bariatric surgery or other surgeries that induce chronic malabsorption |
| 10. | Anti-obesity drugs or diet regimen and unstable body weight three months prior to screening |
| 11. | Treatment with systemic corticosteroids |
| 12. | Blood dyscrasias or any disorders causing hemolysis or unstable red blood cells |
| 13. | Pre-menopausal women who are nursing, pregnant, or of child-bearing potential and not practicing an acceptable method of birth control |
| 14. | Participation in another trial with investigational drug within 30 days of informed consent |
| 15. | Alcohol or drug abuse within three months prior to informed consent that would interfere with trial participation or any ongoing clinical condition that would jeopardize subject safety or study compliance based on investigator judgement |
| 16. | Liver disease, defined by serum levels of alanine transaminase, aspartate transaminase, or alkaline phosphatase >3 x upper limit of normal as determined during screening |
| 17. | Active malignancy at the time of screening |
| 18. | Allergy to iodine-based substances if receiving iohexol for GFR measures |
| Any disqualifying clinical or biochemical parameter may be repeated, at the discretion of the investigator and where there is a clinical reason to do so. The repeat value should be assessed prior to the subject recorded as having screen failed. If the repeat value falls within the ranges defined by the protocol and the patient meets all other inclusion criteria, the patient is eligible for the study. | |

**Supplemental Table S2.** Sodium handling responses to ertugliflozin compared with placebo in participants with type 2 diabetes and heart failure.

|  | **Ertugliflozin (n=17), mean ± SE** | **Placebo (n=17), mean ± SE** | **p-value placebo-adjusted change from baseline** |
| --- | --- | --- | --- |
| FE_Na_, %  Baseline  Week 1  Week 12 | 1.98 ± 0.52  1.42 ± 0.52  1.36 ± 0.48 | 1.53 ± 0.52  1.84 ± 0.52  1.63 ± 0.48 | Ref  0.159  0.157 |
| FE_Li_, %  Baseline  Week 1  Week 12 | 21.06 ± 3.23  21.35 ± 3.23  21.88 ± 3.16 | 21.33 ± 3.23  19.02 ± 3.23  17.64 ± 3.33 | Ref  0.556  0.360 |
| Absolute fractional distal sodium reabsorption, % | | |  |
| Baseline  Week 1  Week 12 | 19.08 ± 3.13  19.93 ± 3.13  20.53 ± 3.06 | 19.79 ± 3.13  17.17 ± 3.13  15.92 ± 3.23 | Ref  0.430  0.263 |
| Relative fractional distal sodium reabsorption | |  |  |
| Baseline  Week 1  Week 12 | 0.889 ± 0.027  0.935 ± 0.027  0.889 ± 0.025 | 0.910 ± 0.027  0.888 ± 0.027  0.910 ± 0.025 | Ref  0.106  0.527 |

Values are in estimated means ± standard error of the mean. FE_Li_ indicates fractional lithium excretion; FE_Na_, fractional sodium excretion. Distal reabsorption is calculated as the difference between FE_Li_ and FE_Na_. Relative fractional distal sodium reabsorption is calculated as the ratio of distal reabsorption and proximal sodium excretion ([FE_Li_-FE_Na_]/ FE_Li_).

Statistical comparisons between placebo and ertugliflozin groups were performed using the Wilcoxon Rank Sum test.

**Supplemental Table S3.** Plasma volume measured by indocyanine green in response to ertugliflozin compared with placebo in participants with type 2 diabetes and heart failure.

|  | **Ertugliflozin (n=9), mean ± SE** | **Placebo (n=9), mean ± SE** | **p-value placebo-adjusted change from baseline** |
| --- | --- | --- | --- |
| Measured plasma volume, ml  Baseline  Week 1  Week 12 | 3711.4 ± 444.7  3845.7 ± 444.7  3470.9 ± 438.0 | 3565.9 ± 500.1  3550.0 ± 500.1  3158.2 ± 491.9 | Ref  0.845  0.849 |

Values are in estimated means ± standard error of the mean. The plasma volume was calculated from the laboratory-measured indocyanine green dye values in *n*=18 participants who had measurements performed.

**Supplemental Table S4.** Safety urine and plasma marker responses to ertugliflozin compared with placebo in participants with type 2 diabetes and heart failure.

|  | **Ertugliflozin (n=17), mean ± SE** | **Placebo**  **(n=17), mean ± SE** | **p-value placebo-adjusted change from baseline** |
| --- | --- | --- | --- |
| Total cholesterol, mmol/l  Baseline  Week 1  Week 12 | 3.56 ± 0.23  3.72 ± 0.23  3.42 ± 0.27 | 3.29 ± 0.23  3.20 ± 0.23  3.43 ± 0.27 | Ref  0.207  0.386 |
| Total protein, g/l  Baseline  Week 1  Week 12 | 70.4 ± 1.3  71.8 ± 1.3  69.9 ± 1.3 | 70.2 ± 1.3  71.1 ± 1.3  72.5 ± 1.4 | Ref  0.601  0.143 |
| Plasma sodium, mmol/l  Baseline  Week 1  Week 12 | 138.8 ± 0.6  139.3 ± 0.6  138.9 ± 0.6 | 139.0 ± 0.6  138.4 ± 0.6  138.3 ± 0.6 | Ref  0.106  0.368 |
| Plasma potassium, mmol/l  Baseline  Week 1  Week 12 | 4.05 ± 0.10  3.98 ± 0.10  4.19 ± 0.13 | 4.03 ± 0.10  4.12 ± 0.10  4.37 ± 0.14 | Ref  0.193  0.423 |
| Plasma phosphate, mmol/l  Baseline  Week 1  Week 12 | 1.05 ± 0.05  1.18 ± 0.05  1.18 ± 0.05 | 0.98 ± 0.05  1.12 ± 0.05  1.04 ± 0.05 | Ref  0.933  0.351 |
| Plasma chloride, mmol/l  Baseline  Week 1  Week 12 | 103.0 ± 0.8  102.2 ± 0.8  103.9 ± 0.8 | 103.8 ± 0.8  103.4 ± 0.8  103.6 ± 0.8 | Ref  0.610  0.305 |
| Plasma calcium, mmol/l  Baseline  Week 1  Week 12 | 2.31 ± 0.06  2.29 ± 0.06  2.28 ± 0.03 | 2.34 ± 0.06  2.23 ± 0.06  2.33 ± 0.03 | Ref  0.471  0.387 |
| Total bilirubin, μmol/l  Baseline  Week 1  Week 12 | 10.1 ± 0.9  8.7 ± 0.9  8.8 ± 1.1 | 8.2 ± 0.9  8.0 ± 0.9  8.0 ± 1.1 | Ref  0.273  0.410 |
| Plasma bicarbonate, mmol/l  Baseline  Week 1  Week 12 | 25.8 ± 0.6  25.8 ± 0.6  24.9 ± 0.7 | 24.2 ± 0.6  24.9 ± 0.6  25.0 ± 0.8 | Ref  0.379  0.135 |
| 24-hour urine albumin, mg/l  Baseline  Week 1  Week 12 | 180.4 ± 72.8  99.7 ± 70.9  110.5 ± 67.4 | 93.2 ± 70.9  98.4 ± 74.8  72.4 ± 68.6 | Ref  0.454  0.480 |
| 24-hour urine protein, g/l  Baseline  Week 1  Week 12 | 0.24 ± 0.07  0.11 ± 0.07  0.20 ± 0.09 | 0.22 ± 0.07  0.19 ± 0.07  0.24 ± 0.09 | Ref  0.340  0.452 |
| 24-hour urine sodium, mmol/l  Baseline  Week 1  Week 12 | 85.2 ± 8.9  81.6 ± 8.7  70.6 ± 7.6 | 80.7 ± 8.9  76.5 ± 8.9  80.7 ± 7.9 | Ref  0.956  0.152 |
| 24-hour urine potassium, mmol/l  Baseline  Week 1  Week 12 | 42.1 ± 3.2  30.5 ± 3.1  33.8 ± 3.0 | 31.9 ± 3.1  25.9 ± 3.2  32.5 ± 3.1 | Ref  0.0003  0.030 |
| 24-hour urine urea, mmol/l  Baseline  Week 1  Week 12 | 218.0 ± 18.3  150.9 ± 18.0  156.4 ± 20.0 | 151.3 ± 18.0  157.3 ± 18.3  142.2 ± 21.6 | Ref  0.0005  0.067 |
